# Decreased DNA density is a better indicator of a nuclear bleb than lamin B loss

**DOI:** 10.1242/jcs.262082

**Published:** 2025-02-11

**Authors:** Samantha Bunner, Kelsey Prince, Emily M. Pujadas Liwag, Nebiyat Eskndir, Karan Srikrishna, Antonia Amonu McCarthy, Anna Kuklinski, Olivia Jackson, Pedro Pellegrino, Shrushti Jagtap, Imuetiyan Eweka, Colman Lawlor, Emma Eastin, Griffin Yas, Julianna Aiello, Nathan LaPointe, Isabelle Schramm von Blucher, Jillian Hardy, Jason Chen, Schuyler Figueroa, Vadim Backman, Anne Janssen, Mary Packard, Katherine Dorfman, Luay Almassalha, Michael Seifu Bahiru, Andrew D. Stephens

**Affiliations:** ^1^ Biology Department, University of Massachusetts Amherst, Amherst, MA 01003, USA; ^2^ Molecular and Cellular Biology, University of Massachusetts Amherst, Amherst, MA 01003, USA; ^3^ Department of Biomedical Engineering, Northwestern University, Evanston, IL 60208, USA; ^4^ IBIS Interdisciplinary Biological Sciences Graduate Program, Northwestern University, Evanston, IL 60208, USA; ^5^ School of Biological Sciences, University of Cambridge, Cambridge CB2 1TN, UK; ^6^ Program in Neuroscience and Behavior, University of Massachusetts, Amherst, MA 01003, USA

**Keywords:** DNA, Bleb, Chromatin, Lamin B, Nucleus

## Abstract

Nuclear blebs are herniations of the nucleus that occur in diseased nuclei and cause nuclear rupture leading to cellular dysfunction. Chromatin and lamins are two of the major structural components of the nucleus that maintain its shape and function, but their relative roles in nuclear blebbing remain elusive. To determine the composition of nuclear blebs, we compared the immunofluorescence intensity of DNA and lamin B in the main nucleus body to that in the nuclear bleb across cell types and perturbations. DNA density in the nuclear bleb was consistently decreased to about half that of the nuclear body whereas lamin B levels in the nuclear bleb varied widely. Partial wave spectroscopic (PWS) microscopy recapitulated the significantly decreased likelihood of high-density domains in the nuclear bleb versus body, and that it was independent of lamin B level. Time-lapse imaging into immunofluorescence revealed that decreased DNA density marked all nuclear blebs whereas decreased lamin B1 levels only occurred in blebs that had recently ruptured. Thus, decreased DNA density is a better marker of a nuclear bleb than lamin B level.

## Teaser

**Highlighted Article:** DNA and lamin B levels in nuclear blebs were measured to determine the best identifying marker; loss of DNA density is a constant for all nuclear blebs across cell type and perturbation whereas lamin B levels vary.

## INTRODUCTION

Nuclear blebs are a hallmark of many human afflictions including aging, progeria, muscular dystrophy, neurological defects and a subset of cancers (Kalukula et al., 2022; Stephens et al., 2019a). An example is prostate cancer where the levels of nuclear blebbing increase with pathology grade measured as Gleason Score (Helfand et al., 2012). Studies over the past 20 years have deduced the nuclear components that are essential for maintaining normal ellipsoidal nuclear shape are chromatin epigenetic modifications (Furusawa et al., 2015; Schreiner et al., 2015; Stephens et al., 2018, 2019b), lamins (Chen et al., 2018; Lammerding et al., 2006; Vahabikashi et al., 2022), and linkers within and between them (Belaghzal et al., 2021; Jung-Garcia et al., 2023; Liu et al., 2021; Strom et al., 2021). Upon perturbation of these components the nucleus becomes weaker and is deformed by external forces from actin contraction (Jung-Garcia et al., 2023; Nmezi et al., 2019; Pho et al., 2024a), actin compression (Hatch and Hetzer, 2016; Le Berre et al., 2012), transcription activity (Berg et al., 2023) and/or migration through constrictions (Denais et al., 2016; Pfeifer et al., 2018; Raab et al., 2016). This deformation of a normally elliptical nucleus leads to a herniation called a nuclear bleb that forms during interphase (Hatch and Hetzer, 2016; Stephens et al., 2019b; Vargas et al., 2012). The high curvature of this bleb leads to nuclear rupture that then causes nuclear dysfunction via disruption of DNA damage and repair pathways, transcription and cell cycle control (Kalukula et al., 2022; Stephens, 2020; Xia et al., 2018). However, the composition of a nuclear bleb remains highly debated and elusive. A hindrance has been the ability to differentiate interphase-forming nuclear blebs from mitosis-forming micronuclei, which can appear similar (Krupina et al., 2021). A clear determination of nuclear bleb composition will provide insights into how a nuclear bleb forms and how to accurately detect it.

The nuclear lamina is composed of four separate but interacting peripheral meshworks of lamin intermediate filaments – A, C, B1 and B2 (Shimi et al., 2015; Turgay et al., 2017). It has been widely reported that nuclear blebs are devoid of both B-type lamins via qualitative data, whereas quantitative data disagree. Some publications go as far as using loss of lamin B1 to determine what is and is not a nuclear bleb by renaming blebs lamin B-less domains (LBLDs; Bercht Pfleghaar et al., 2015; Helfand et al., 2012). Many publications provide qualitative data via one to three example images to conclude that nuclear blebs are devoid of lamin B (Bercht Pfleghaar et al., 2015; Cho et al., 2018; Gauthier and Comaills, 2021; Kamikawa et al., 2021; Patteson et al., 2019; Shimi et al., 2008; Wren et al., 2012; Yang et al., 2005). Recent publications have begun to quantify lamin B1 levels in nuclear blebs revealing that 25–50% of nuclear blebs are actually positive for lamin B (Jung-Garcia et al., 2023; Nmezi et al., 2019; Stephens et al., 2018). Thus, statements made using qualitative single images are not supported by quantitative measurements of lamin B1 levels in nuclear blebs. Instead, recent quantitative studies point to a more complicated picture of lamin B1 levels, which can vary widely in nuclear blebs. We hypothesize that the disconnected nature of these studies, each working on one cell type and perturbation, leads to an incomplete view of nuclear bleb composition. Thus, nuclear bleb composition needs to be quantitatively analyzed across perturbations, cell types, and live-cell and disease models.

Decreased DNA density in the nuclear bleb provides an alternative indicator of nuclear blebs compared to lamin B. In contrast to the mixed reporting of lamin B, nuclear blebs are more consistently reported to have less DNA in the bleb. Furthermore, enrichment of euchromatic markers has been qualitatively and quantitatively reported in nuclear blebs (Shimi et al., 2008; Stephens et al., 2018). Whereas decreased DNA and histone modification decompaction are reported more consistently in nuclear blebs, a rigorous study across multiple perturbations and cell lines is required to determine the consistency of DNA nuclear bleb labeling compared to lamin B1.

To determine the composition of nuclear blebs, we first devised a strategy to differentiate nuclear blebs from micronuclei using line scans and live-cell imaging of DNA via SiR-DNA staining. We used immunofluorescence to label DNA and lamin B1 or B2 in mouse and human cells to compare the levels in the nuclear bleb to the main nuclear body. Mouse embryonic fibroblasts (MEFs) provide the ability to compare the composition of wild type (WT) low-percentage nuclear blebbing to chromatin or lamin perturbations previously reported to increase nuclear blebbing (Kalukula et al., 2022; Stephens et al., 2019a). In MEFs, DNA density displayed a consistent decrease in the nuclear bleb across WT and perturbations whereas whether the lamin B presence in the bleb was altered significantly depended on the condition. Live-cell imaging of nuclear blebs and nuclear bleb formation using CRISPR-labeled human HCT116 GFP–lamin B1 cells revealed inconsistent lamin B presence in the bleb whereas SiR-DNA density was consistently decreased. Next, we compared human disease cell models of progeria and prostate cancer, which revealed yet again decreased DNA density in the nuclear bleb whereas lamin B1 was absent in progeria blebs and present in prostate cancer nuclear blebs. Finally, we utilized live-cell partial wave spectroscopic (PWS) microscopy to confirm decreased DNA density in nuclear blebs. Finally, time-lapse imaging into immunofluorescence revealed that lamin B levels are dependent on nuclear rupture or lack thereof. Our results indicate that DNA density is a more reliable marker for nuclear blebs than lamin B across multiple perturbations and cell types.

## RESULTS

### Nuclear blebs can be differentiated from micronuclei

**Fig. 1.**
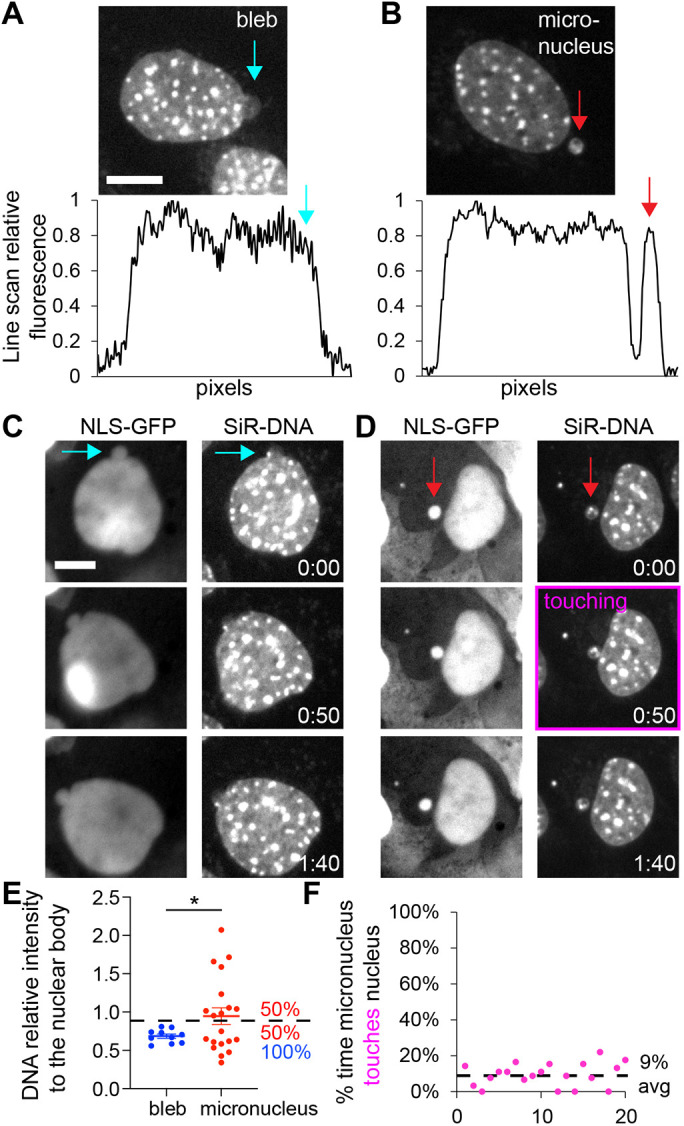
**Nuclear blebs and micronuclei can be reliably differentiated through quantitative measurements.** (A,B) Example images of SiR-DNA labeled WT MEF nuclei with a (A) nuclear bleb or (B) micronucleus. Line scans of relative fluorescence intensity of the example images show attachment for nuclear blebs (blue arrow) and separation (red arrow) for micronuclei from the nuclear body. (C,D) Example time-lapse imaging of MEF nuclei via NLS–GFP and SiR-DNA for a (C) blebbed nucleus (blue arrow) and (D) micronucleus (red arrow). The purple box highlights when the micronucleus touches the nucleus. Time stamps are hours:minutes. (E) Graph quantifying SiR-DNA intensity in the nuclear bleb (blue, *n*=10) and micronucleus (red, mean±s.e.m. *n*=20) relative to the nuclear body. Dashed line denotes 0.9 relative intensity and percentage of events above and below this line are noted in respective colors. (F) Graph of the percentage of time the micronucleus touches the nucleus over a 3-h period for *n*=20 micronuclei. Dashed line denotes the mean percentage. *P<0.05; no asterisk denotes no significance, *P*>0.05 (two-tail unpaired Student's *t*-test). Scale bars: 10 µm.

Nuclear blebs and micronuclei are different phenomena that can appear similar. To determine the composition of nuclear blebs, we developed clear parameters to distinguish nuclear blebs from micronuclei. Nuclear blebs are defined as herniations of the main nucleus that form during interphase (Stephens et al., 2018, 2019b; Vargas et al., 2012). By contrast, a micronucleus is separate from the main nuclear body and forms due to mitotic segregation and reformation failures (Krupina et al., 2021). In MEFs, nuclear blebs and micronuclei can be differentiated in static images via a line scan, where micronuclei are separate from the main nucleus (Fig. 1A,B). For rigorous verification, we used live-cell time-lapse imaging of nuclear localization signal-tagged green fluorescence protein (NLS–GFP) and SiR-DNA to show that nuclear blebs move with the nuclear body over time whereas micronuclei move separately (Fig. 1C,D). However, during time-lapse imaging, micronuclei can touch the main nuclear body which might cause a micronucleus to appear similar to a nuclear bleb by line scan (Fig. 1D, purple outline). First, we measured DNA density in nuclear blebs and micronuclei to determine possible differences. Relative to the main nuclear body, nuclear blebs show a consistently decreased DNA density<0.8 with an average of 0.68±0.03 (mean±s.e.m.). Conversely, micronuclei show a significantly higher and more variable DNA density where 50% present a greater concentration than the nucleus and 50% are less concentrated, averaging 0.96±0.14 (*P*<0.05, Fig. 1E). Similarly, in human HCT116 and prostate cancer cell lines LNCaP and DU145, micronuclei showed higher DNA density compared to nuclear blebs (two-tailed unpaired Student's *t*-test *P*<0.05, Fig. S1A). Micronuclei were infrequent in both LNCaP and PC3. Next, we aimed to assess the probability of a static image presenting a micronucleus touching the nucleus that could be mistaken for a nuclear bleb. Tracking 20 micronuclei in MEFs over 3 h at 2-min intervals revealed that micronuclei touch the nucleus 9% of the time (Fig. 1F). Thus, our approach outlines a methodology for separating nuclear blebs from micronuclei for static images in a manner that has a low failure rate.

To verify that Hoechst 33342 stain is quantitative of DNA density, we imaged FUCCI HT1080 nuclei that provide a clear reporter of diploid G1 nuclei via fluorescent Cdt1 and tetraploid late S/G2 nuclei via fluorescent Geminin (Marcus et al., 2015). Hoechst 33342 sum intensity doubled from diploid G1 to tetraploid late S/G2 nuclei alongside the near doubling of nucleus size (Fig. S1B–D), supporting that Hoechst 33342 intensity is quantitative of relative DNA density in agreement with previous reports (Chiu et al., 2024; Kim and Sederstrom, 2015). Both human HT1080 fibrosarcoma cells and RPE-1 retinal pigment epithelial cells show decreased DNA density in nuclear blebs relative to the nuclear body (Fig. S1E,F), recapitulating MEF nuclear bleb data. Finally, staining with a different DNA dye propidium iodide in MEFs showed a similar decrease of DNA density to 0.5±0.05 in the nuclear bleb relative to the body whereas micronuclei showed a significant increase to 1.4±0.13 (Fig. S1G). Thus, decreased DNA dye intensity in nuclear blebs reports a quantitative significantly decreased DNA density across multiple mammalian cell lines.

### Nuclear blebs have consistently decreased DNA density whereas lamin B1 levels are more variable and change significantly depending on condition

**Fig. 2.**
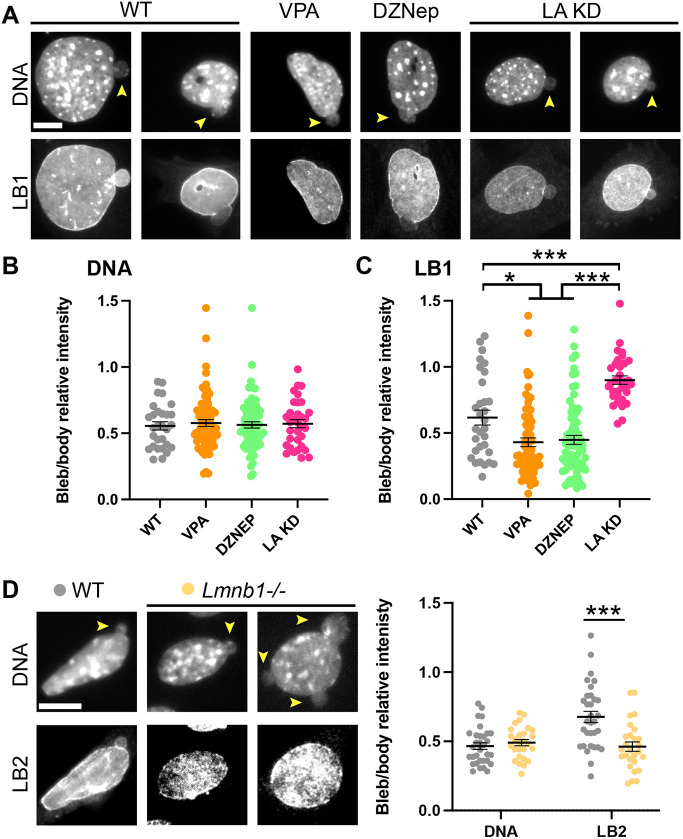
**Nuclear blebs show consistently decreased DNA density whereas lamin B1 is more variable and changes based on perturbation.** (A) Example images of MEF blebbed nuclei from WT, after chromatin decompaction overnight drug treatments to increase euchromatin levels (valproic acid; VPA) or decrease heterochromatin (DZNep), and lamin A KD (LA KD). Nuclei were labeled with DAPI to stain DNA, anti-lamin B1 immunofluorescence (LB1). (B,C) Graphs of bleb-to-body relative intensity ratio for (B) DNA and (C) LB1 for WT *n*=31, VPA *n*=67, DZNep *n*=71, and LA KD *n*=33. (D) Example images and graph of blebbed nuclei in MEF WT (*n*=32) and lamin B1 knockout (*Lmnb1*^−/−^, *n*=26) labeled with DAPI to stain DNA and anti-lamin B2 immunofluorescence (LB2). Graph shows bleb-to-body relative intensity ratio for DNA and LB1. Yellow arrowheads denote nuclear blebs. Mean±s.e.m. is graphed. *P<0.05, ***P<0.001, no asterisk denotes no significance, *P*>0.05 (multiple two-tail unpaired Student's *t*-test). Scale bars: 10 µm.

To compare the composition of nuclear blebs and their consistency between WT cells and those with perturbations that induce increased nuclear blebbing, we used MEFs. Nuclear blebs were determined by the presence of a >1 µm protrusion of the nucleus as previously defined (Berg et al., 2023; Eskndir et al., 2024; Pho et al., 2024a; Stephens et al., 2018, 2019b). To determine the composition of nuclear blebs, we applied immunofluorescence imaging to label nuclei for DNA (DAPI) and lamin B1 or B2. We measured the relative levels of each component within the nuclear bleb and compared it to the main nuclear area, referred to as the nuclear body. In WT MEF cells the mean intensity measurements revealed a significantly decreased DNA density in the nuclear bleb relative to that in the body to give 0.55±0.03 (mean±s.e.m.) (Fig. 2A,B). Lamin B1 staining also revealed a significant decrease in levels in the nuclear bleb compared to the body to 0.62±0.06. However, ratios of fluorescent signals in the nuclear bleb to the body were ∼2-fold more variable than DNA (average deviation DNA 0.13 versus lamin B1 0.26, Levene's test *P*=0.006; Fig. 2A–C). This was largely due to 26% of nuclear blebs showing no loss of lamin B1, whereas all measured nuclear blebs show a decrease of DNA (no loss is >0.9 relative intensity, *n*=31), in agreement with previous reports (Stephens et al., 2018). Overall, WT nuclear blebs present an overall loss of both DNA and lamin B1 in nuclear blebs.

Chromatin decompaction perturbations cause increased nuclear blebbing without changes in nuclear lamins (Stephens et al., 2018). Applying these perturbations provides an opportunity to determine whether non-lamin-based nuclear perturbation causes any changes in bleb composition relative to WT cells. Specifically, we used the histone deacetylase inhibitor valproic acid (VPA) which increases euchromatin (Llères et al., 2009; Yankulov et al., 1995) and the histone methyltransferase inhibitor DZNep to decrease levels of heterochromatin (Miranda et al., 2009; Stephens et al., 2018). Both treatments that caused chromatin decompaction-induced nuclear blebs also showed consistently decreased levels of DNA in the nuclear bleb relative to the body, similar to that of nuclear nuclear blebs in WT (Fig. 2A,B). On the other hand, chromatin decompaction-induced nuclear blebs showed a significant decrease in lamin B1 staining relative to WT nuclear blebs (WT 0.62 versus VPA 0.43 and DZNep 0.45±0.03, *P*<0.05, Fig. 2A,C). Thus, chromatin decompaction results in similar decreased levels of DNA in the nuclear bleb compared to in the nuclear body to what is seen in WT, but a greater decrease in lamin B1 levels in the bleb compared to in the body.

To further characterize the composition of these nuclear blebs and determine the most consistent marker for them, we repeated these experiments on nuclear blebs induced by lamin perturbation. Lamin A is usually present in nuclear blebs after nuclear rupture, when it is recruited, but is absent in pre-ruptured blebs (Denais et al., 2016; Kamikawa et al., 2021; Sears and Roux, 2022; Shimi et al., 2008, 2015). Loss of lamin A alone, as achieved by constitutive shRNA-mediated knockdown (KD), increases nuclear blebbing (Berg et al., 2023; Pho et al., 2024b; Vahabikashi et al., 2022), and provides the ability to determine whether lamin A presence or absence affects the composition of the nuclear bleb. Lamin A KD-induced nuclear blebs showed a similar significant loss of DNA signal in the bleb compared to the body as in WT cells (Fig. 2A,B). Interestingly, lamin A KD-induced nuclear blebs showed a drastic increase in lamin B1 bleb-to-body ratio compared to WT nuclear blebs (WT 0.62 versus LA KD 0.90±0.03, *P*<0.001, Fig. 2A,B). Thus, lamin A KD blebs showed on average no significant loss of lamin B1 in the nuclear bleb relative to the nuclear body (*P*>0.05). Taken together, nuclear blebs in WT, and chromatin perturbation- and lamin A KD-induced nuclear blebs have similar levels of decreased DNA density in the bleb whereas each condition has a significantly different level of lamin B1 in nuclear blebs.

### Lamin B2 levels are variable in nuclear blebs and can be altered by perturbations while DNA density in nuclear bleb remains consistent

Lamin B1 loss was one of the first reported perturbations that results in increased nuclear blebbing (Hatch and Hetzer, 2016; Lammerding et al., 2006; Shimi et al., 2015; Vargas et al., 2012). To determine whether lamin B1 loss alters the composition of the nuclear blebs, we applied immunofluorescence imaging to label DNA and lamin B2 in both WT and lamin B1-knockout (*Lmnb1^−/−^*) MEF cells. As above, DNA density was consistently decreased in nuclear blebs relative to the body in WT cells (0.48±0.03, mean±s.e.m., Fig. 2D). As was the case for other nuclear perturbations that increased nuclear blebbing, lamin B1-knockout cells also displayed a relatively consistent decrease in DNA in the nuclear bleb relative to the nuclear body, similar to WT (0.49**±**0.02, Fig. 2D). Thus, decreased DNA density in nuclear blebs was measured consistently for WT and all chromatin and lamin perturbations.

Next, we looked at lamin B2 presence in nuclear blebs. Like lamin B1, lamin B2 levels in nuclear blebs also varied more than DNA in WT cells (average deviation DNA 0.14 versus lamin B2 0.21, Levene's test *P*<0.05; Fig. 2D). In lamin B1-knockout cells, lamin B2 displayed a significant decrease in nuclear blebs relative to WT (WT 0.69±0.05 versus *Lmnb1^−/−^* 0.47±0.03, *P*<0.001, Fig. 2D). These data suggest that although nuclear blebs show a clear and significant loss of DNA density across perturbations, the levels of lamin B2 are dependent on lamin B1 (Fig. 2D), which we show is dependent on both histone modification state and lamin A levels (Fig. 2A–C).

### Live-cell imaging of nuclear blebs recapitulates the consistent loss of DNA and variable lamin B1 presence

**Fig. 3.**
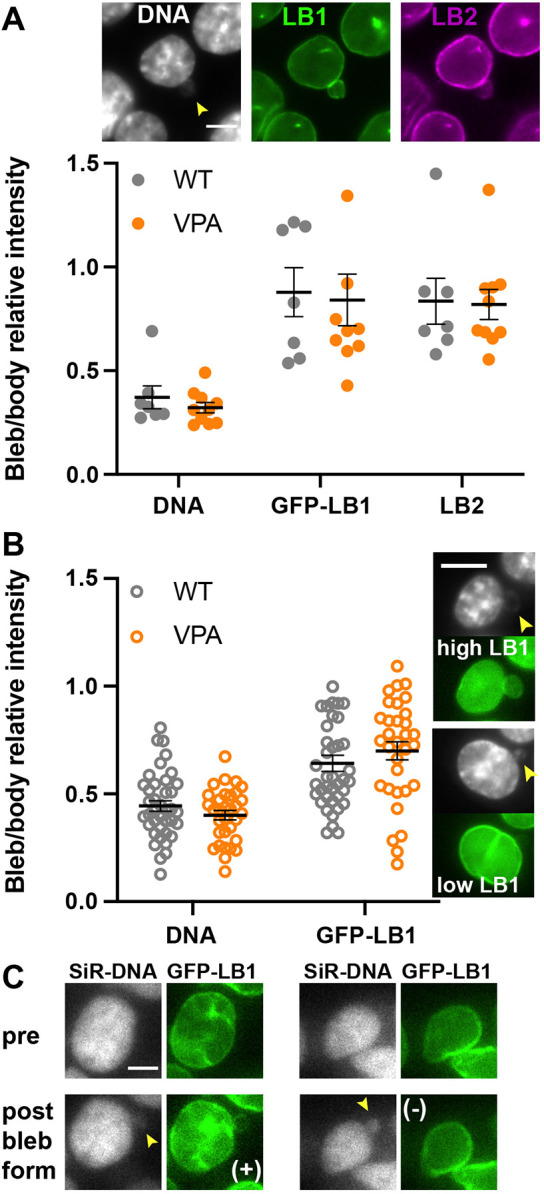
**Live-cell imaging consistently reproduces decreased DNA and variable lamin B1 in nuclear blebs.** (A) Example image and graph of relative levels in the nuclear bleb determined by immunofluorescence imaging of HCT116 cells with CRISPR labeled GFP-lamin B1 with Hoechst-labeled DNA and anti-lamin B2 labeling in untreated (*n*=7) and VPA-treated (*n*=10) HCT116 cells. (B) Graph and example images of live-cell imaging levels of Hoechst-stained DNA and GFP–lamin B1 in the nuclear bleb relative to in the nuclear body in untreated (*n*=39) and VPA-treated (*n*=32) from biological triplicates. (C) Example images from live-cell timelapse imaging of nuclear bleb formation in HCT116 GFP–lamin B1 cells with DNA stained by SiR-DNA showing that nuclear blebs can form either with similar levels of lamin B1 (+) or lower levels of lamin B1 (−). Yellow arrowheads denote nuclear blebs. Mean±s.e.m. is graphed. Scale bars: 5 µm.

To further explore nuclear bleb composition, we used HCT116 colon cancer cells with a CRISPR-labeled endogenous GFP–lamin B1. First, we performed immunofluorescence staining for both DNA (DAPI) and lamin B2 to compare with GFP–lamin B1. Similar to the experiments with MEFs, we investigated rare nuclear blebbing in WT cells and increased nuclear blebbing via VPA-treatment to decompact chromatin. Immunofluorescence imaging recapitulated the major findings from MEFs as DNA density was consistently decreased in nuclear blebs relative to the nuclear body (two-tailed unpaired Student's *t*-test *P*<0.05) while both lamin B1 and B2 levels did not (two-tailed unpaired Student's *t*-test *P*>0.05, Fig. 3A). Interestingly, while VPA-treated MEF nuclei show a decrease in lamin B1 bleb levels compared to WT (Fig. 2B), this significant decrease of lamin B1 upon VPA does not occur in HCT116 (Fig. 3). This data further highlights the inconsistent and variable labeling of nuclear blebs by lamin B. To verify that fixed-cell immunofluorescence does not cause artifacts, we next imaged nuclear bleb composition in live cells. Live-cell imaging of GFP–lamin B1 and Hoechst 33342-stained DNA revealed that both WT and VPA-treated nuclear blebs presented a consistent decreased level of DNA density in the nuclear bleb relative to the body whereas the lamin B1 level did not significantly change (Fig. 3B), a recapitulation of the data obtained via fixed-cell immunofluorescence. Furthermore, lamin B1 levels in the nuclear bleb relative to the body were more variable than DNA density in both WT and VPA-treated cells (average deviation for DNA of 0.10 and 0.12, versus for lamin B1 of 0.18 and 0.19, respectively, Levene's test *P*<0.05; Fig. 3B). Both MEF and human HCT116 nuclear blebs show a clear and consistent loss of DNA density whereas lamin B levels vary.

Studies have postulated that localized loss of lamin B1 aids nuclear bleb formation (Lammerding et al., 2006). Along these lines, lamin B1 loss could be temporary during nuclear bleb formation, which might lead to newly formed nuclear blebs having no lamin B1 whereas long-lived nuclear blebs do have lamin B1. To test this hypothesis, we live-cell imaged nuclear bleb formation in HCT116 GFP–lamin B1 cells with SiR-DNA. We found that nuclear blebs formed with high and low levels of lamin B1, suggesting that the variance of lamin B1 levels in nuclear blebs occurs during formation (Fig. 3C). Even in live-cell imaging of nuclear bleb formation, SiR-DNA was decreased in the nuclear bleb upon formation. Taken together, imaging of nuclear blebs via fixed immunofluorescence, and static live-cell, and dynamic live-cell imaging during bleb formation show that there is a consistent loss of DNA density in blebs whereas bleb lamin B1 levels are highly variable.

### Different cell types and models of human disease have a similar loss of DNA density in the nuclear bleb, whereas lamin B1 levels change drastically

Based on the consistent results we obtained in MEFs and HCT116 cells, we next tested the reliability of decreased DNA as a marker for blebbing in different disease models that are associated with increased nuclear deformation and blebbing – human progeria disease and prostate cancer cell lines. Progeria syndromes are rare genetic disorders that recapitulate many aspects of normal aging and are mainly caused by mutations in DNA repair proteins or proteins associated with the nuclear envelope (Butin-Israeli et al., 2012)**.** Furthermore, loss of lamin B1 in nuclear blebs has frequently been reported in progeria disease model cell lines (Bercht Pfleghaar et al., 2015; Cho et al., 2018; Gauthier and Comaills, 2021; Shimi et al., 2008; Yang et al., 2005). The use of prostate cancer cell line models provides the ability to assay lamin B1 presence in a different model of human disease.

**Fig. 4.**
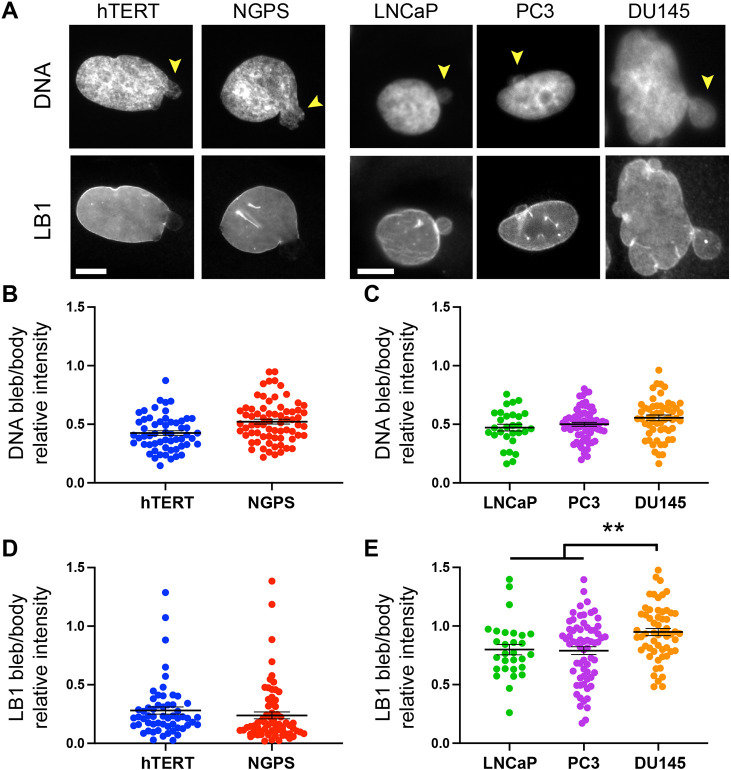
**Progeria and prostate cancer model cell lines recapitulate decreased DNA density in nuclear blebs whereas lamin B1 drastically differs.** (A) Example images of a blebbed nucleus in hTERT and NGPS human fibroblasts along with human prostate cancer model cell lines LNCaP, PC3 and DU145. Nuclei are labeled with DAPI to stain DNA and anti-lamin B1 immunofluorescence (LB1). Yellow arrowheads denote nuclear blebs. (B,C) Graphs of bleb-to-body relative intensity ratio for (B) DNA and (C) LB1 for human WT (hTERT, *n*=57) and progeria (NGPS, *n*=70) fibroblasts from biological triplicates. (D,E) Graphs of bleb-to-body relative intensity ratio for (D) DNA and (E) LB1 human prostate cancer cells of increasing aggressiveness. LNCaP, *n*=39; PC3, *n*=68; DU145, *n*=57 all from biological triplicates. Mean±s.e.m. is graphed. **P<0.01; no asterisk denotes no significance, *P*>0.05 (two-tailed unpaired Student's *t*-test). Scale bars: 10 µm.

To determine quantitative levels of lamin B1 in progeria-based nuclear blebs, we used WT (hTERT) and Nestor–Guillermo progeria syndrome (NGPS) immortalized human fibroblasts to measure the relative DAPI and lamin B1 levels in bleb. NGPS is caused by a homozygous A12T point mutation in barrier-to-autointegration-factor 1 (BAF; also known as BANF1), a small protein that binds to DNA, A-type lamins and several nuclear envelope proteins (Cabanillas et al., 2011; Puente et al., 2011). The disease mutation decreases the interaction between A-type lamins and BAF (Janssen et al., 2022b) resulting in a lack of A-type lamin recruitment to nuclear envelope ruptures. Cells from individuals with NGPS had blebs that have thus been reported to lack A-type lamins (Janssen et al., 2022b). Analysis of DNA density consistently showed that there was significantly decreased DNA labeling intensity in the nuclear bleb relative to the nuclear body whereas not changing between control (hTERT) and progeria (NGPS) human fibroblasts (Fig. 4A,B) or prostate cancer cell lines (Fig. 4A,C). Conversely, lamin B1 levels varied drastically between the two different model cell lines (two-tailed unpaired Student's *t*-test *P*<0.001, Fig. 4D,E). Lamin B1 was drastically decreased in the majority of nuclear blebs compared to the levels in the nuclear body in both WT (hTERT) and NGPS fibroblasts (Fig. 4A,D), suggesting that lamin B1 loss in nuclear blebs is not specifically due to the BAF mutation. In most prostate cancer cells, lamin B1 was present in nuclear blebs at similar levels to the rest of the nucleus (Fig. 4E). However, a minor subpopulation of cells did show near complete loss in some blebs. Interestingly, different stages of prostate cancer do show significant changes in lamin B1 levels in nuclear blebs. The less-aggressive LNCaP and moderately aggressive PC3 prostate cancer cell lines showed variable loss of lamin B1 levels in nuclear blebs resulting in a bleb-to-body ratio of ∼0.75±0.04 (mean±s.e.m., Fig. 4D). The more-aggressive DU145 showed no loss of lamin B1 in nuclear blebs (0.95) making them significantly different from LNCaP and PC3 (Fig. 4E). Overall, lamin B1 levels in nuclear blebs in hTERT WT and NGPS cells were drastically lower than all prostate cancer cell lines, which showed high levels of lamin B1 in nuclear blebs (Fig. 4D versus E, *P*<0.001). The higher level of lamin B1 in the nuclear bleb was not due to confusion of micronuclei as a bleb because micronuclei were extremely rare in live-cell imaged NLS–GFP LNCaP (2/326 cells, 0.6%) and PC3 fixed cells (63 blebs versus four micronuclei, Fig. 4; Fig. S1A). These results confirm not only that loss of DNA density is a reliable indicator across different cell types, but also that between cell types and certain disease models lamin B1 levels in nuclear blebs change significantly.

### Loss of DNA density is a consistent metric of nuclear blebbing across perturbations

Finally, we assessed whether the results obtained with immunofluorescence imaging would still hold using an alternative high-throughput technique that can detect statistical changes in chromatin structure across entire cell populations. PWS microscopy provides label-free measurements of nanoscale structural changes with a sensitivity to structures between ∼20 and ∼200 nm in live cells without the use of exogenous labels (Almassalha et al., 2016; Gladstein et al., 2018, 2019). To do this, PWS directly measures variations in spectral light interference that results from light scattering. As this scattering is due to heterogeneities in chromatin density (Li et al., 2021), PWS provides a way to confirm that reduced DNA density is a reliable marker for nuclear blebbing. Chromatin packing scaling (D) can be calculated from these fluctuations in chromatin density. To obtain D measurements within nuclear blebs and nuclear bodies, we used HCT116 cells modified with the auxin-inducible degron (AID) system to induce simultaneous rapid lamin B1 and lamin B2 degradation (Nishimura et al., 2009; Pujadas Liwag et al., 2024; Yesbolatova et al., 2019), termed HCT116^LMN(B1&B2)-AID^ cells.

**Fig. 5.**
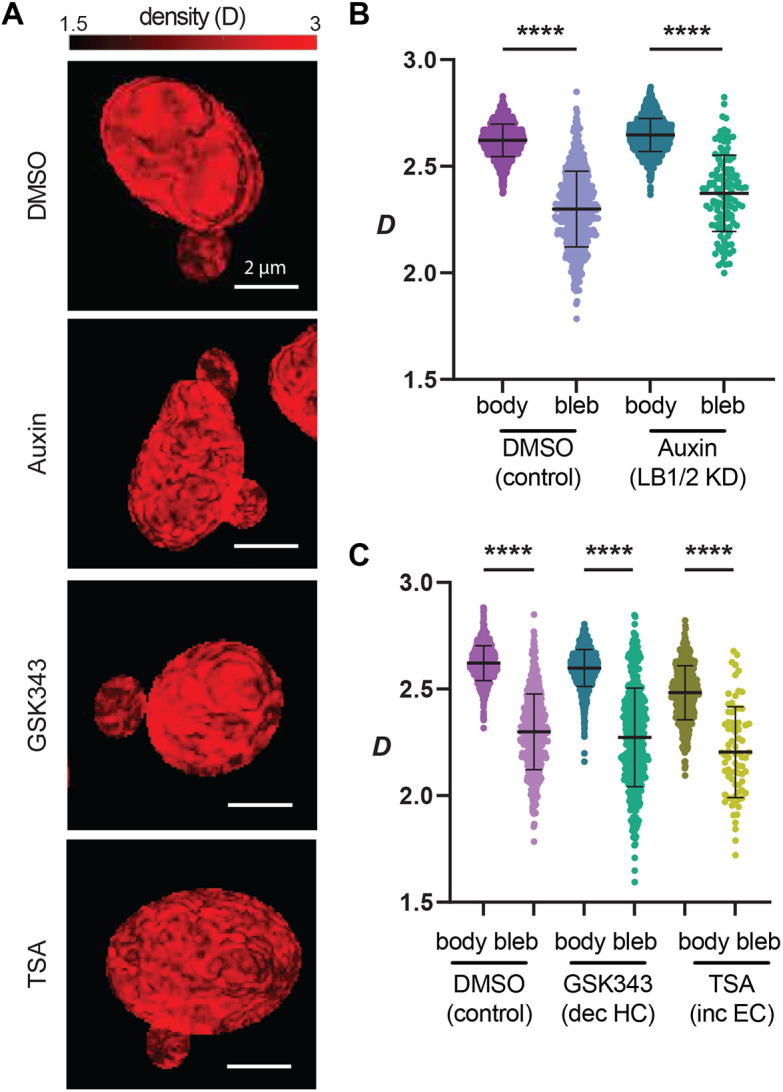
**PWS reveals a consistent loss of DNA density in nuclear blebs.** (A) Representative PWS D maps for each treatment type in HCT116^LMN(B1&B2)-AID^ cells. Scale bars: 2 µm. (B) Live-cell PWS microscopy demonstrates that the mean D is much lower in the nuclear bleb than the nuclear body in untreated (control) and auxin-treated cells (LB1/2 KD) (control nuclear body, *n*=2437 and blebs, *n*=564; auxin-treated nuclear body, *n*=2105 and blebs, *n*=129). (C) Live-cell PWS microscopy demonstrates that the mean D is much lower in the nuclear bleb than the nuclear body upon GSK343 (control nuclear body, *n*=775 and blebs, *n*=466) or TSA treatment (control nuclear body, *n*=496 and blebs, *n*=77) compared to the 24 h DMSO treatment (vehicle control, nuclear body *n*=740 and blebs *n*=458). Error bars show the median and interquartile ranges with all data points behind. ****P≤0.0001 (unpaired two-tailed Student's *t*-test between selected groups).

Using PWS, we confirmed that HCT116^LMN(B1&B2)-AID^ cell nuclei showed a significant decrease in D in the nuclear bleb relative to the nuclear body (Fig. 5), similar to the observed decrease in Hoechst 33342-labeled fluorescence measures seen in HCT116 (Fig. 3). This decrease in D was shown for both control and auxin-induced simultaneous degradation of lamin B1 and lamin B2 (Fig. 5A,B). To confirm these results in cells with decompacted chromatin, we then treated HCT116^LMN(B1&B2)-AID^ cells with GSK343, a potent EZH2 inhibitor that specifically prevents H3K27 methylation (Verma et al., 2012), and Trichostatin A (TSA), a specific HDAC class I and II inhibitor (Yoshida et al., 1990). Our results demonstrated significant decreases in D within nuclear blebs of DMSO-treated control, GSK343-treated, and TSA-treated cells in comparison to nuclear main nuclear body (Fig. 5C). These differences were apparent in representative D maps of each condition (Fig. 5A). These results demonstrate that in live-cells, either with or without the presence of B-type lamins, with EZH2 inhibition, and with HDAC inhibition, reduced DNA or chromatin density is a consistent marker of nuclear blebbing. Taken together, this work establishes a more reliable method of quantifying and characterizing nuclear blebs across multiple perturbations and cell types. We therefore provide a more complete assessment of nuclear bleb composition.

### Lamin B1 levels are dependent on nuclear bleb integrity whereas DNA density is independent of it

**Fig. 6.**
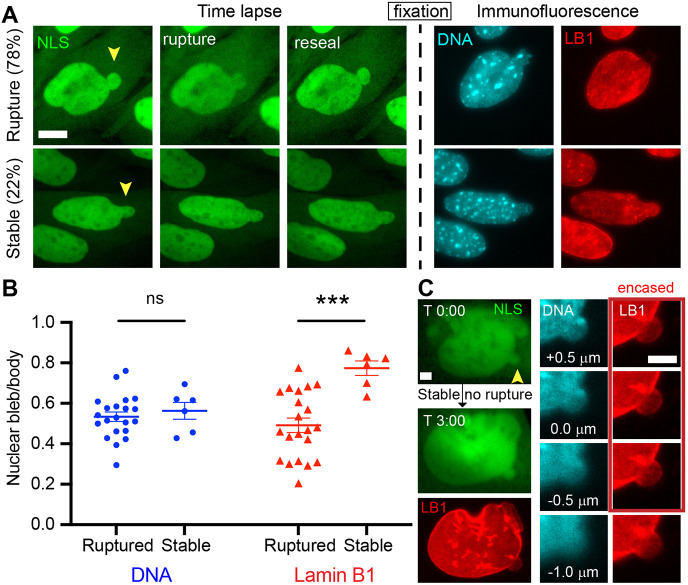
**Time-lapse imaging of nuclear integrity followed by immunofluorescence measurements reveals recent nuclear rupture decreases lamin B1 levels but does not change DNA density.** (A) Representative MEF NLS–GFP time-lapse images followed by fixed immunofluorescence imaging of DNA and lamin B1 (LB1) in the same nucleus. Top shows an example of a nucleus that ruptures <30 min before fixed immunofluorescence whereas bottom shows stable non-ruptured nuclear bleb. (B) Graph of nuclear bleb intensity normalized to the nuclear body intensity for DNA and lamin B1 in ruptured and stable nuclei (*n*=27 nuclei). Mean±s.e.m. is graphed. ***P<0.001; ns, no significance, *P*>0.05 (two-tailed unpaired Student's *t*-test). (C) Example stable nuclear bleb that does not rupture over 3 h and is verified by time lapse. Right panels show magnified *z*-stacks of the nuclear bleb DNA (cyan) and lamin B1 (red) shows that the bleb is encased in lamin B1 but is also clearly connected to the nuclear body. Images in C are from a single representative example. Yellow arrowheads denote nuclear blebs. Scale bars:10 µm.

Levels of other nuclear envelope components are affected by nuclear rupture and repair (Gunn et al., 2024; Halfmann et al., 2019; Young et al., 2020). We hypothesized that lamin B1 level variability in the nuclear bleb across different cell lines and perturbations could be dependent on bleb integrity. To assay lamin B1 levels in nuclear blebs of nuclei that rupture or are stable, we time-lapse imaged live MEF NLS–GFP cells on a gridded dish then immediately fixed and conducted immunofluorescence of DNA and lamin B1. Time-lapse imaging over 3 h at 3-min intervals revealed that nuclei with nuclear blebs either ruptured, spilling NLS–GFP and resealing in 10 min, or remained stable (Fig. 6A). Immunofluorescence of these same nuclei revealed that DNA density remained similar between ruptured and stable nuclear blebs, suggesting DNA density is unaffected by nuclear rupture. Conversely, lamin B1 levels in recently ruptured nuclear blebs was significantly less than nuclear blebs that remained stable and did not rupture (0.49±0.04 ruptured versus 0.77±0.04 stable, *P*<0.05, Fig. 6B). Furthermore, time-lapse-verified nuclear blebs showed encirclement of the nuclear bleb by lamin B1 throughout most of the confocal *z*-stack, but also show clear connectedness of the bleb to the nuclear body (Fig. 6C). Thus, lamin B1 levels can change significantly between ruptured and stable blebs providing a mechanism for the variability between cell types and perturbations.

## DISCUSSION

Nuclear blebs are both hallmarks of and a contributor to cellular dysfunction in human diseases. Even though their importance is clear, we still understand very little about nuclear blebs. A striking example is the lack of understanding around nuclear bleb composition and a consistent marker for nuclear blebs. Previous, qualitative reports focused on a few images showing a loss of lamin B in nuclear blebs. However, data standards now require quantification, which revealed that lamin B levels in nuclear blebs varies widely. In this report, we used fluorescence imaging to quantify the levels of both DNA and lamin B1 in nuclear blebs across four different cell types and many more perturbations. We provide quantitative data showing how drastically lamin B1 levels can differ in nuclear blebs due to either cell type or perturbation and more specifically that it is dependent on nuclear bleb rupture. By contrast, we find that, relative to the main nucleus, nuclear blebs consistently display decreased DNA density across cell types, perturbations and levels of disease progression. Our analysis reveals that these findings are consistent between different imaging modalities by using PWS to measure the statistical chromatin density. Our work provides a change in the paradigm of how nuclear blebs might be quantitatively identified through DNA density and not by lamin B1 levels.

### The ever-growing importance of DNA and chromatin in nuclear blebbing

Chromatin is now a well-established determinant of nuclear blebs (Furusawa et al., 2015; Kalinin et al., 2021; Stephens et al., 2018, 2019b) and overall nuclear shape (Schibler et al., 2023; Schreiner et al., 2015; Tamashunas et al., 2020). Isolated single-nucleus micromanipulation force measurements provide the ability to separate chromatin and lamin mechanical contribution to nuclear force resistance (Currey et al., 2022; Stephens et al., 2017), which was recapitulated by AFM-LS (atomic force microscopy combined with side-view light sheet imaging) (Hobson et al., 2020). Chromatin acts as the initial and main mechanical component of the nucleus that resists the antagonistic external cytoskeletal forces (Pho et al., 2024a) and internal transcriptional chromatin movement (Berg et al., 2023) that drive nuclear blebbing, which leads to nuclear rupture and dysfunction (Kalukula et al., 2022; Stephens, 2020; Stephens et al., 2019a). Modeling of nuclear mechanics and morphology show strong support for the importance of chromatin (Banigan et al., 2017; Berg et al., 2023; Hobson and Stephens, 2020; Liu et al., 2021). Thus, it is not surprising that decreased DNA density provides a strong indicator of nuclear blebs.

Overall, decreased DNA density in the nuclear bleb provides the most consistent indicator to date. Decreased levels of DNA in the nuclear bleb is consistent across different cell types, multiple perturbations and levels of disease progression. More specifically, decreased concentration of DNA in the nuclear blebs was demonstrated in both untreated WT cells, which show a low percentage of nuclear blebbing, and upon perturbation by chromatin decompaction (by VPA, TSA, DZNep and GSK343) or lamin perturbations (LA KD, *Lmnb1^−/−^* and GFP–AID–lamin B1), which display a high percentage of nuclear blebbing (Fig. 2). This suggests that decreased DNA density is a hallmark of nuclear blebbing that is independent of both global histone modification state and lamins. Thus, these data support the idea that DNA and/or chromatin decreased density is an essential factor driving nuclear bleb formation. At the least, nuclear bleb formation is not dependent on lamin B1 composition as example time-lapse imaging shows that nuclear blebs form with or without lamin B1 (Fig. 3C). This same data showed a decreased level of DNA in the nuclear bleb relative to the nuclear body. Finally, there was also no effect on DNA density in nuclear blebs between WT and progeria (NGPS) fibroblasts and less-aggressive (LNCaP) and more-aggressive (DU145) prostate cancer cells. Taken together, the consistency of decreased DNA density in nuclear blebs strongly supports chromatin decompaction as being a driving mechanism in nuclear bleb formation.

### Lamin B levels in nuclear blebs are highly dependent on cell type, perturbation and, most directly, nuclear rupture

Previous studies of nuclear bleb composition in progeria have been pivotal in building our current understanding of this important phenomena, which is both a hallmark and cause of human disease. Several groups have reported that, across different progeria types, lamin B1 and B2 are absent in nuclear blebs based on qualitative data (Bercht Pfleghaar et al., 2015; Butin-Israeli et al., 2012; Shimi et al., 2008). Our data suggests that lamin B loss reported in previous papers focused on progeria models is likely due to the use of human fibroblasts, which quantitatively show little to no lamin B in nuclear blebs (Fig. 4). It is important to note that previous qualitative data are supported by quantitative data, but that the generalization of lamin B loss to other cell types and/or perturbations in blebs is not supported. This data is heavily contrasted by our data for mouse fibroblasts, human colon cancer cells (HCT116), and many prostate cancer cells (LNCaP, PC3 and DU145), which display varied or unchanged levels of lamin B in nuclear blebs (Figs 2–4). Furthermore, we reveal that lamin B1 levels in the nuclear bleb can be altered based on chromatin or lamin perturbation (Fig. 2). Thus, many quantitative studies of nuclear bleb composition now show that lamin B1 levels can vary widely and depend on cell type and perturbation (Janssen et al., 2022a,b; Jung-Garcia et al., 2023; Nmezi et al., 2019; Stephens et al., 2018).

The changes in lamin B levels in nuclear bleb are most directly affected by nuclear integrity. Nuclear blebs are sites of high curvature that are well documented to lead to nuclear rupture (Xia et al., 2018). Time-lapse imaging into immunofluorescence provides valuable insight into lamin B variable levels by showing that recently ruptured nuclear blebs have decreased levels whereas stable blebs that have not recently ruptured have similar levels of lamin B1 relative to the nuclear body (Fig. 6). This finding is also supported by other parts of our work. Lamin B levels in the nuclear bleb were lower in cells with perturbations (VPA, DZNep and *Lmnb1*^−/−^) than in WT cells (Fig. 2). These perturbations have been reported to increase in the frequency of rupture for blebbed nuclei by 2-fold or more (Pho et al., 2024a; Stephens et al., 2019b; Vargas et al., 2012; Young et al., 2020). The rupture frequency of lamin A KD nuclear blebs has not been reported, as this is a relatively new tool (Vahabikashi et al., 2022), but we would hypothesize the lack of lamin B1 loss in the bleb relative to the body is due to lack of bleb rupture. Similarly, we would hypothesize both hTRET and NGPS progeria nuclear blebs rupture frequently causing them to present low lamin B1 levels in the nuclear bleb. Conversely, we would hypothesize nuclear blebs in the prostate cancer cells lines rupture infrequently, which results in their high levels of lamin B1 in the bleb (Fig. 4). Future work will be required to determine whether lamin B level variation in blebs across cells and perturbations is due to bleb rupture or stability.

### Nuclear bleb composition informs possible models of nuclear bleb formation and rupture

The composition of the nuclear bleb might aid in uncovering the steps during nuclear bleb formation, a process that remains elusive. There are a few theoretical models for nuclear bleb formation when considering very simple parameters of chromatin and lamins. Chromatin could simply push out the nuclear lamina/envelope and maintain similar levels of lamin proteins. This theoretical model has data to support it in that nuclear blebs with high lamin B1 levels appear to have a completely intact lamina around the main nuclear body and the nuclear bleb (Figs 2–4). Data from time-lapse imaging support that this first model would not result in nuclear rupture, but instead a stable bleb (Fig. 6). A slight deviation of this idea is that chromatin could flow through a small hole, or holes, in the lamina resulting in pushing out some lamins and other nuclear envelope proteins but not others. Flowing of chromatin into the bleb is supported by PWS and super-resolution imaging showing fragmentation of heterochromatin into nanoscopic domains in the bleb (Pujadas Liwag et al., 2025). This model could result in a decreased level of lamin proteins but not complete loss. This model is partially supported by data showing that, upon formation, nuclear blebs can have varying levels of lamin B1 (Fig. 3) but it would require more fine-tuned experiments to support it as a driving model. This second model could produce sufficient thinning of the nuclear envelope with increased strain to break the nuclear envelope resulting in nuclear rupture (Fig. 6). Overall, the two theoretical models of nuclear bleb formation relying on chromatin as a driving force are partially supported by experimental data and might best explain stable blebs that do not rupture (first model) versus blebs that rupture (second model).

A third model of nuclear bleb formation is that the lamina breaks or has a hole through which chromatin flows to form the nuclear bleb. Previous reports have noted that lamin B1 gaps in the nuclear lamina could allow for chromatin to flow out to form a nuclear bleb. We provide multiple pieces of data to refute this model. First, HCT116 nuclear blebs can form with or without endogenously labeled lamin B1 (Fig. 3) clearly showing loss of lamin B1 is not required whereas decreased DNA density is consistent. Second, nuclear blebbing increases similarly in cells with chromatin perturbations, lamin B1 perturbations and lamin A KD (Berg et al., 2023; Vahabikashi et al., 2022). If local lamin B loss drove nuclear bleb formation, lamin B perturbations would be expected to result in greater levels of nuclear blebbing, but the data does not support this (Berg et al., 2023; Pho et al., 2024a; Stephens et al., 2018, 2019b). The data presented here show that lamin A KD shows no significant loss of lamin B1 from nuclear blebs (Fig. 2C), directly contradicting this third model. The data that previously supported this third model was that lamin B1-negative nuclear blebs appear to have a sizeable gap between the nuclear body lamina and the nuclear bleb devoid of lamin B. Our novel data show that the loss of lamin B1 from the bleb making a clear ‘hole’ is most likely the result of a recent rupture (Fig. 6). Thus, drastic loss or a gap of lamin B1 is not a precursor to or cause of bleb formation but instead is the result or consequence of nuclear envelope rupture at the bleb. Taken together, our data supports the idea that many different theoretical models of chromatin and lamin interactions can arise to form a nuclear bleb of various lamin compositions, but the only uniting mechanism is lower DNA density.

### Future directions

Nuclear bleb composition, formation and consequences remain a valuable topic for discovery. This work has deduced the consistency of using DNA to label nuclear blebs, underlying its importance in nuclear bleb formation. However, many questions remain surrounding histone modification states that dictate chromatin compaction. Can investigating nuclear bleb composition inform the role of euchromatin, facultative heterochromatin and constitutive heterochromatin in nuclear bleb formation? Our findings on the variable presence of lamin B in nuclear blebs raise many questions about other nuclear lamina and envelope proteins such as lamin A and C, nuclear pore complexes (NPCs), emerin, BAF, LAP2, LBR and many more. The major next step is to track nuclear bleb composition alongside dynamic events of nuclear envelope rupture and repair and nuclear bleb formation. Recent studies have shown that BAF, LEMD2 and emerin are recruited to nuclear envelope ruptures (Gunn et al., 2024; Halfmann et al., 2019; Young et al., 2020). We provide data showing that lamin B1 levels are dependent on nuclear bleb integrity. Future studies could bridge these two approaches to show the temporal behavior of known nuclear envelope repair proteins and lamin B1 composition relative to nuclear rupture events tracked by NLS–GFP spilling. This advancement could provide the ability to determine rupture dynamics in static images. Through leveraging our understanding of nuclear bleb composition, it is possible to gain a deeper understanding of both nuclear bleb formation and rupture, a prominent event in human disease.

## MATERIALS AND METHODS

### Cell culture

MEFs were as previously described (Shimi et al., 2008; Stephens et al., 2018; Vahabikashi et al., 2022). MEF wild-type (WT), lamin A KD (LA KD), and lamin B1 knockout (*Lmnb1*^−/−^) cells were cultured in DMEM (Corning) completed with 10% fetal bovine serum (FBS, HyClone) and 1% penicillin-streptomycin (Corning). Cells were grown and incubated at 37°C and 5% CO_2_, passaged every 2 to 3 days. Human fibrosarcoma cell HT1080 cells were cultured and passaged similarly. Human RPE-1 retinal pigment epithelial cells were cultured with a DMEM/F12 medium (Corning, 10-090-CV) and passaged similarly.

Both HT1080 and RPE-1 cells were obtained from the Orth laboratory (University of Colorado Boulder, USA) and had a fluorescent ubiquitin cell cycle indicator (FUCCI) with lentiviral plasmids mKO2-hCdt1(30/120) (DDBJ/EMBL/GenBank, AB370332) in pCSII-EF vector and mAG-hGem(1/110) (DDBJ/EMBL/GenBank, AB370333) in pCSII-EF.

Three prostate cancer cell lines were used: LNCaP, DU145 and PC3. DU145 and LNCaP cells were cultured in RPMI 1640 (Corning) with 10% FBS and 1% penicillin-streptomycin. PC3 cells were cultured in DMEM completed with 10% FBS and 1% penicillin.

HCT116^LMN(B1&B2)-AID^ cells were grown in McCoy's 5A Modified Medium (#16600-082, Thermo Fisher Scientific) supplemented with 10% FBS (#16000-044, Thermo Fisher Scientific) and 100 μg/ml penicillin-streptomycin (#15140-122, Thermo Fisher Scientific). To create these cells, HCT116 cells (ATCC, #CCL-247) were tagged with the AID system as previously described (Pujadas Liwag et al., 2024). All cells were cultured under recommended conditions at 37°C and 5% CO_2_. All cells in this study were maintained between passage 5 and 20. Cells were allowed at least 24 h to re-adhere and recover from trypsin-induced detachment. All imaging was performed when the surface confluence of the dish was between 40–70%. All cells were tested for mycoplasma contamination (ATCC, #30-1012K) before starting perturbation experiments, and they have given negative results.

NGPS cells (NGPS5787) and control fibroblasts (AG10803) were immortalized with SV40LT and TERT. These immortalized cell lines were a gift from Carlos López-Otín (Universidad de Oviedo, Asturias, Spain) and were cultured in DMEM containing 10% fetal bovine serum and 1% penicillin-streptomycin. Cells were maintained at 37°C and 5% CO_2_.

All cell lines were tested for contamination weekly and were obtained from ATCC, unless stated otherwise, or authenticated before beginning experiments.

### Biochemical treatments

MEF WT cells were treated with either 4 mM valproic acid (VPA, 1069-66-5, Sigma) or 1 µM 3-deazaneplanocin (DZNep; Miranda et al., 2009) for 24 h before fixation and immunofluorescence.

HCT116^LMN(B1&B2)-AID^ cells were plated at 50,000 cells per well of a 6-well plate (Cellvis, P12-1.5H-N). To induce expression of OsTIR1, 2 μg/ml of doxycycline (Thermo Fisher Scientific, #10592-13-9) was added to cells 24 h prior to auxin treatment. 1000 μM indole-3-acetic acid sodium salt (IAA, Sigma-Aldrich, #6505-45-9) was solubilized in RNase-free water (Thermo Fisher Scientific, #10-977-015) before each treatment as a fresh solution and added to HCT116^LMN(B1&B2)-AID^ cells.

HCT116^LMN(B1&B2)-AID-GFP^ cells were plated at 50,000 cells per well of a 6-well plate (Cellvis, P12-1.5H-N). Cells were given at least 24 h to re-adhere before treatment. VPA was dissolved in medium and cells were treated with 4 mM. GSK343 (Millipore Sigma, #SML0766) was dissolved in DMSO to create a 10 mM stock solution. This was further diluted in complete cell media to a final treatment concentration of 10 µM. TSA (Millipore Sigma, #T1952) was diluted in complete cell medium and added to cells at a final treatment concentration of 300 nM.

### Live-cell time-lapse fluorescence imaging

As previously described, we used either established HCT116^LMN(B1&B2)-AID-GFP^ cells or MEF NLS–GFP stable cell lines to quantify nuclear shape and rupture (Berg et al., 2023; Pho et al., 2024a). For tracking live-cell DNA density, cells were treated with 1 µm SiR-DNA (Cytoskeleton, CY-SC007) and 1 µm verapamil (Cytoskeleton, CY-SCV01) 1 h before imaging. Images were acquired with Nikon Elements software on a Nikon Instruments Ti2-E microscope, Orca Fusion Gen III camera, Lumencor Aura III light engine, TMC CLeanBench air table, with a 40× air objective (NA 0.75, W.D. 0.66, MRH00401). Live-cell time-lapse imaging was possible using the Nikon Perfect Focus System and a Okolab heat, humidity and CO_2_ stage top incubator (H301). Cells were imaged in either 4-well cover glass dishes or 8-well cover glass chambers (Cellvis). For time-lapse data, images were taken in 2-min intervals during 3 h with six fields of view for each condition single plane.

### Immunofluorescence

Cells were grown on coverslips in preparation. Cells were fixed with 3.2% paraformaldehyde and 0.1% glutaraldehyde in phosphate-buffered saline (PBS) for 10 min. Between steps cells were washed with three times PBS with 0.1% Tween 20 and 0.2 g/l azide (denoted PBS-Tw-Az). Next, cells were permeabilized by 0.5% Triton-X 100 in PBS for 10 min. Again, cells were washed with PBS-Tw-Az. Humidity chambers were prepared using Petri dishes, filter paper, distilled water and parafilm. 50 µl of primary antibody solution was applied to the parafilm and the cell-side and the coverslip was placed on top to incubate for 1 h at 37°C in the humidity chamber. Primary antibodies used were mouse monoclonal anti-nuclear pore complex proteins at 1:1000 (Mab414, Abcam ab24609), rabbit polyclonal anti-lamin B1 at 1:1000 (Abcam ab16048) and rabbit recombinant monoclonal anti-lamin B2 at 1:1000 (Abcam ab151735). The humid chambers were removed from the incubator and the coverslips were washed in PBS-Tw-Az. More humidity chambers were made for the secondary incubation. 50 µl of secondary antibody was placed on the parafilm, and the coverslips were placed cell-side down. The secondary antibody solution contained goat anti-mouse antibodies FITCI at 1:1000 dilution and goat anti-rabbit dilution TRITCI at 1:1000 dilution (from Cell Signaling Technologies) and incubated in humidity chambers at 37°C for 30 min. After, coverslips were washed with PBS-Tw-Az. The coverslips were placed cell-side down on a slide with a drop of mounting media containing DAPI. Alternatively, cells were stained with a 1 µg/ml (1:10,000) dilution of Hoechst 33342 (Life Technologies) in PBS for 5 min and then washed with PBS three times and mounted with Prolong Fade gold. Slides were allowed to cure for 4 days at 4°C before imaging.

As previously described (Janssen et al., 2022a,b), hTERT and NGPS cells were fixed at room temperature for 10 min with 4% PFA. Cells were washed in PBS, permeabilized using 0.2% Triton X-100, and blocked using 2% bovine serum albumin (BSA) in PBS for 30 min. Cells were incubated overnight at 4°C or for 1 h at room temperature (RT) in 2% BSA PBS containing primary antibody mouse anti-lamin B1 (Santa Cruz Biotechnology, sc-365214, 1:500). Cells were washed using PBS and incubated for 1 h at room temperature with secondary antibody in 2% BSA PBS. Cells were washed in PBS and mounted using Prolong Gold (Thermo Fisher Scientific). Images were taken on Zeiss Axio Imager Z2 using a 63× oil immersion objective (Plan APO, NA 1.4, Zeiss).

For live-cell imaging into immunofluorescence experiments we used a gridded imaging dish (Cellvis #D35-14-1.5GI). Transmitted images were acquired to align the gridded dish after fixation so that the same cells from the time lapse imaging could be imaged for their respective immunofluorescence of DNA via Hoechst 33342 and lamin B1.

### Immunofluorescence imaging

Immunofluorescence images were acquired using a QICAM Fast 1394 Cooled Digital Camera, 12-bit, Monochrome CCD camera (4.65×4.65 µm pixel size and 1.4 MP, 1392×1040 pixels) using Micromanager and a 40× objective lens on a Nikon TE2000 inverted widefield fluorescence microscope. Cells were imaged using transmitted light to find the optimal focus on the field of view to observe the nuclear blebs. Ultraviolet light (excitation 360 nm) was used to visualize DNA via DAPI, blue light (excitation 480 nm) was used to visualize nuclear pore complex (NPC) and green light (excitation 560 nm) was used to visualize lamin B1 or B2. Images were saved and transferred to Fiji imaging software for analysis (Schindelin et al., 2012).

### Nuclear bleb analysis

Images were exported to Fiji imaging software (Schindelin et al., 2012) to analyze the intensity of each component normalizing bleb intensity to the nuclear body intensity. The body, bleb and background of each nucleus image were measured by drawing regions of interest (ROIs) via the polygon selection tool. The researcher performing this analysis was aware of the experimental conditions. Measurements of mean intensity for each ROI were recorded and exported to Excel. Within Excel, the background intensity was subtracted from the body and bleb. Then the average bleb intensity was divided by the average nuclear body intensity to give a relative measure, where the same average intensity would result in 1. These values were transferred to Prism where Mann–Whitney test, unpaired two-tailed *t*-tests, or one way ANOVA with two posts test were performed, and the data was graphed. All raw data is provided in Table S1.

### PWS image acquisition and analysis

For live-cell measurements, cells were imaged and maintained under physiological conditions (5% CO_2_ and 37°C) using a stage-top incubator (In Vivo Scientific, Salem, SC; Stage Top Systems). Live-cell PWS measurements were obtained using a commercial inverted microscope (Leica, DMIRB) using a Hamamatsu Image-EM charge-coupled device (CCD) camera (C9100-13) coupled to a liquid crystal tunable filter (LCTF, CRi VariSpec) to acquire monochromatic, spectrally resolved images ranging from 500–700 nm at 2-nm intervals as previously described (Almassalha et al., 2016; Gladstein et al., 2018, 2019). Broadband illumination was provided by a broad-spectrum white light LED source (Xcite-120 LED, Excelitas). The system is equipped with a long-pass filter (Semrock BLP01-405R-25) and a 63× oil immersion objective (Leica HCX PL APO). All cells were given at least 24 h to re-adhere before treatment (for treated cells) and imaging. Briefly, PWS measures the spectral interference signal resulting from internal light scattering originating from nuclear chromatin. This is related to variations in the refractive index (RI) distribution (Σ) (extracted by calculating the standard deviation of the spectral interference at each pixel), characterized by the chromatin packing scaling (D). D was calculated using maps of Σ, as previously described (Eid et al., 2020; Gladstein et al., 2019; Virk et al., 2020). Measurements were normalized by the reflectance of the glass medium interface (i.e. to an independent reference measurement acquired in a region lacking cells on the dish). This allows us to obtain the interference signal directly related to RI fluctuations within the cell. Although it is a diffraction-limited imaging technique, PWS can measure chromatin density variations because the RI is proportional to the local density of macromolecules (e.g. DNA, RNA, proteins). Therefore, the standard deviation of the RI (Σ) is proportional to nanoscale density variations and can be used to characterize packing scaling behavior of chromatin domains with length scale sensitivity around 20–200 nm, depending on sample thickness and height. Changes in D resulting from each condition are quantified by averaging cells, taken across three technical replicates. Average D was calculated by first averaging D values from PWS measurements within each cell nucleus and then averaging these measurements over the entire cell population for each treatment condition.

## Supplementary Material



10.1242/joces.262082_sup1 Supplementary information

Table S1. Raw data. The raw data values from each figure are contained in this excel document.
