# Supplementary material for: Decreased DNA density is a better indicator of a nuclear bleb than lamin B loss
Source: J Cell Sci. 2025 Feb 11;138(3):jcs262082. doi: 10.1242/jcs.262082 (PMC11883270; doi:10.1242/jcs.262082)
Supplement: Supplementary information [file joces-138-262082-s1.pdf]

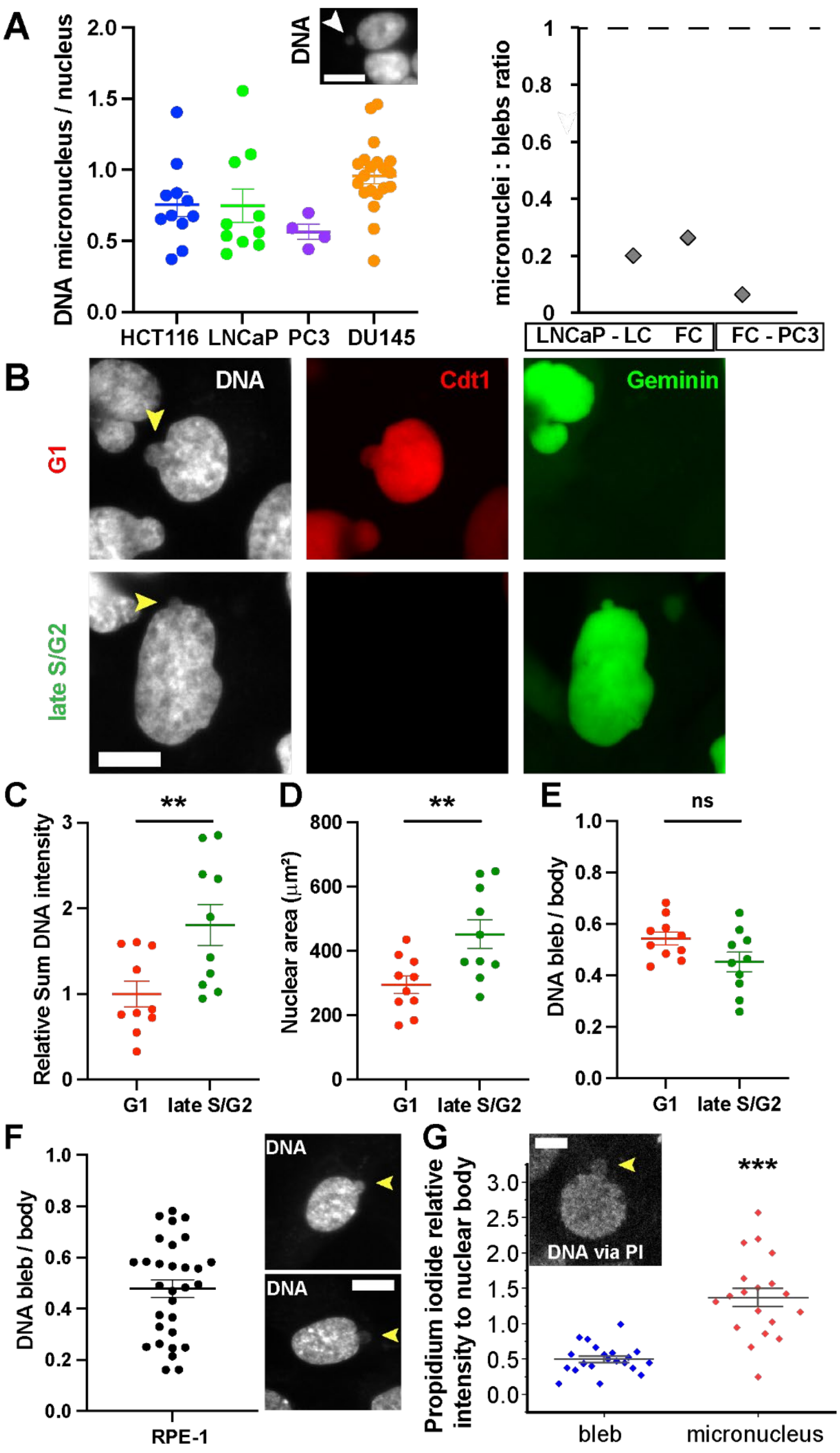

Journal of Cell Science • Supplementary information

Fig. S1. Continued quantification of DNA in micronuclei and nuclear blebs. (A) Left panel: HCT116, LNCaP, and DU145 micronuclei measure significantly higher levels of DNA density relative to the nuclear body than nuclear blebs (t-test $p < 0.05$ compared to bleb data in main Figures, micronuclei n = 11, 10, 4, 20 respectively). Example image of HCT116 micronucleus. Scale bars represent 2 μm. Right panel: Graph of the ratio of micronuclei to nuclear blebs for LNCaP live cell (LC) via NLS-GFP imaging over 3 hours and fixed cell (FC) via DAPI in both LNCaP and PC3. Micronuclei were rare relative to blebs in all cases respectively (n= 2/10, 10/38, and 4/63). (B). Example images of HT1080 nuclei with FUCCI markers. Graphs of G1 vs. late S/G2 (C) relative DNA sum intensity, (D) nuclear area, and (E) DNA bleb intensity relative to nuclear body for n = 10 for each. (F) Graph of RPE-1 DNA bleb intensity relative to nuclear body and example images (n = 20 nuclei). (G) Graph of propidium iodide relative intensity of nuclear blebs (n = 21) and micronuclei (n = 19) to the nuclear body. This data is similar to SiR-DNA in Fig. 1E. Propidium iodide staining was accomplished by RNase treatment before staining with PI, use 2X SSC (0.3 M NaCl,0.03 M sodium citrate, pH 7.0). Incubate the sample in 100 ug/mL of DNase-free RNase in 2X SSC for 20 minutes. Wash 3 times for 1 min in 2X SSC. Then use PI in a 1:3,000 dilution with 2X SSC. Add 300 uL per well and incubate for 1-5 min. Repeat the same rinse process as before then mount. Statistical tests were determined by two-tail unpaired Student's t-test p values reported as * < 0.05, ** < 0.01, *** < 0.001. Mean ± s.e.m is graphed. Scale bar = 10 μm.

Table S1. Raw data. The raw data values from each figure are contained in this excel document.

Available for download at
https://journals.biologists.com/jcs/article-lookup/doi/10.1242/jcs.262082#supplementary-data
