# Supplementary material for: Decreased DNA density is a better indicator of a nuclear bleb than lamin B loss
Source: J Cell Sci. 2025 Feb 11;138(3):jcs262082. doi: 10.1242/jcs.262082 (PMC11883270; doi:10.1242/jcs.262082)
Supplement: Supplementary file 1 [file joces-138-262082_review_history.pdf]

Decreased DNA density is a better indicator of a nuclear bleb than lamin B loss

Samantha Bunner, Kelsey Prince, Emily M Pujadas Liwag, Nebiyat Eskndir, Karan Srikrishna, Pedro Pellegrino, Julianna Aiello, Antonia Amonu McCarthy, Colman Lawlor, Shrushti Jagtap, Anna Kuklinski, Emma Eastin, Griffin Yas, Nathan LaPointe, Olivia Jackson, Imuetiyan Eweka, Isabelle Schramm von Blucher, Jillian Hardy, Jason Chen, Schuyler Figueroa, Vadim Backman, Anne Janssen, Mary Packard, Katherine Dorfman, Luay Almassalha, Michael Seifu Bahiru and Andrew D Stephens
DOI: 10.1242/jcs.262082

Editor: Guillaume Jacquemet

Review timeline

Original submission:	1 March 2024
Editorial decision:	16 April 2024
First revision received:	15 August 2024
Editorial decision:	10 September 2024
Second revision received:	28 October 2024
Accepted:	30 October 2024

Original submission

First decision letter

MS ID#: JOCES/2024/262082

MS TITLE: Decreased DNA density is a better indicator of a nuclear bleb than lamin B loss

AUTHORS: Samantha Bunner, Kelsey Prince, Karan Srikrishna, Emily M Pujadas Liwag, Pedro Pellegrino, Julianna Aiello, Antonia Amonu McCarthy, Colman Lawlor, Shrushti Jagtap, Anna Kuklinski, Griffin Yas, Nathan LaPointe, Imuetiyan Eweka, Emma Eastin, Olivia Jackson, Jason Chen, Isabelle Schramm von Blucher, Jillian Hardy, Vadim Backman, Anne Janssen, Mary Packard, Katherine Dorfman, Luay Almassalha, Michael Seifu Bahiru, and Andrew D Stephens
ARTICLE TYPE: Research Article

We have now reached a decision on the above manuscript.

To see the reviewers' reports and a copy of this decision letter, please go to: https://submit-jcs.biologists.org and click on the 'Manuscripts with Decisions' queue in the Author Area. (Corresponding author only has access to reviews.)

As you will see, the reviewers raise a number of substantial criticisms that prevent me from accepting the paper at this stage. They suggest, however, that a revised version might prove acceptable, if you can address their concerns. If you think that you can deal satisfactorily with the criticisms on revision, I would be pleased to see a revised manuscript. We would then return it to the reviewers.

Please ensure that you clearly highlight all changes made in the revised manuscript. Please avoid using 'Tracked changes' in Word files as these are lost in PDF conversion.

© 2024. Published by The Company of Biologists under the terms of the Creative Commons Attribution License (https://creativecommons.org/licenses/by/4.0/).

I should be grateful if you would also provide a point-by-point response detailing how you have dealt with the points raised by the reviewers in the 'Response to Reviewers' box. Please attend to all of the reviewers' comments. If you do not agree with any of their criticisms or suggestions please explain clearly why this is so.

Reviewer 1

Advance summary and potential significance to field

In this manuscript Bunner et al. characterize DNA labeling density versus lamin B labeling on nuclear blebs arising in multiple cell lines and conditions including in response to altered chromatin states, nuclear lamina organization and cancer types, and find that decreased DNA density is a more robust indication of a nuclear bleb than loss of lamins or nuclear pore complexes (NPCs).

This is a necessary study that seeks to clarify a persistent issue in the field with identifying and classifying nuclear morphology changes and provides a new tool for researchers to accurately quantify nuclear blebs. The authors take several approaches to validate their approach, including assessing protein and DNA intensity in fixed and live cells and by partial wave spectroscopy. Each approach shows a consistent decrease in DNA density in nuclear blebs across multiple conditions. This study could be of significant interest to researchers studying nuclear envelope structure, nuclear mechanics and cancer cell biology. A major limitation is that it does not clarify how nuclear blebs are distinguished from a major contaminating object class – micronuclei - and it is not clear that the widefield imaging system used has the power to do so.

Comments for the author

I would support the publication of this manuscript if the following points can be addressed:

Major Points

1. Both micronuclei and nuclear blebs can appear as round DNA positive and often lamin positive objects abutting the nucleus. Although some images of lamin B1 positive blebs show a clear continuation between the DNA or lamin in the nucleus in the bleb (e.g. Fig. 1A, WT, bleb LB1+), as expected, in many cases such continuity is not visible or noticeably absent in this class of objects (e.g. Fig. 1A LA KD bleb LB1+, left; Fig. 1D WT; Fig. 2A; Fig. 3A LNCaP). In contrast continuity of the DNA is easily visible in all of the LB1- examples. In addition, the live-cell imaging example of a lamin B1 positive bleb forming (Fig. 2C), which would be very conclusive that blebbing is a major mechanism of lamin B1/DNA positive object formation, shows an extruded object with much lower levels of lamin B1 than in many of the example images, consistent with multiple classes of objects being included in the lamin B1+ category.

A critical part of the bleb definition is that the DNA and nucleoplasm are continuous with the nucleus. With the widefield imaging set up available this could mean identifying focal planes for all the images where the DAPI appears to change intensity gradually between the nucleus and bleb, which could be visualized in a line scan. Ideally the authors would repeat these analyses on a confocal or deconvolution system with a nuclear envelope marker that would allow them to show that the blebs have a continuous rim of lamin B1 with the nucleus. In addition, the authors should induce large micronuclei in these cells, for instance through nocodazole arrest or inhibition of the mitotic checkpoint, to determine how frequently large micronuclei have decreased DNA intensity and normal lamin B1 labeling in their cell lines. Although recent work suggests that micronuclear DNA is compact and that lamin levels can be very low there may be significant differences across cell lines and conditions.

2. The NPC antibody used in figure 1A has too much background to determine its localization at the nucleus periphery. Although 414 is a standard NPC marker Nup133 and Nup153 are also common enumeration labels that frequently give a cleaner signal than 414.

Minor point

© 2024. Published by The Company of Biologists under the terms of the Creative Commons Attribution License (https://creativecommons.org/licenses/by/4.0/).

1. The resolution of Fig. 4 is significantly lower than the rest making it harder to read and see what's going on in the nucleus renderings.

Reviewer 2

Advance summary and potential significance to field

This is an interesting work addressing the question of the composition of the nuclear blebs, with the goal of defining the correct marker of a nuclear bleb.
Though this is an important point in the biology of blebs and would be useful for the scientific community in the field, there are several issues that weaken authors' conclusions.

Comments for the author

Below my specific comments that the authors could address to improve their manuscript in a revised version.
One of the main problems with the presented results is that it is often unclear if the authors are considering real nuclear blebs or micronuclei that stay close to the surface of the main nucleus. This is an important point, because micronuclei could have lamina, and this could be the reason why the authors observe variability in all the studied cell lines in the presence/absence of lamin B1 in the supposed nuclear blebs. The authors should discuss this. Another problem is the methods used to quantify DNA as the measurement of the fluorescence intensity of DAPI, Hoechst and siRDNA is not commonly considered reliable. Both issues undermine the validity of authors' conclusions.
Specific points:
- Abstract- " Chromatin has been reported to be decreased or decompacted in nuclear blebs": the authors are probably referring to heterochromatin and not generally chromatin that is decreased, they should rephrase the sentence.
-Introduction- "Studies over the last 20 years have deduced the nuclear components that are essential for maintaining normal ellipsoidal nuclear shape are chromatin…": the authors should be more specific and mention that the epigenetic modifications play an essential role in this regulating chromatin compaction degree as discussed in recent reviews.
- Introduction- The authors should mention the difference between nuclear blebs and micronuclei, which is an important piece of the puzzle they want to address in their manuscript. A distinction between the two phenotypes could be indeed important to unravel the question of the real composition of the nuclear blebs as reported previously (Utani et al. PlosONE 2010, 2011).
-Results- "To determine the composition of nuclear blebs, we applied immunofluorescence imaging to label nuclei for DNA (DAPI) …"- DAPI staining is not usually considered suitable for quantitative measurements of DNA, among other things it only binds A-T rich sequences, as such the results the authors are showing are not conclusive nor indicative. However, it is true that there is in literature an indication that reducing DAPI concentration it could be used for quantification by cytofluorimetry, but the authors did not mention the concentration of DAPI they used, nor the parameters used at the microscope to acquire the DAPI images, instead it would seem that they simply used a common mounting media containing DAPI as reported in the methods section. In previous publications the authors used this method (DAPI/Hoechst fluorescence intensity) to quantify DNA, however, considering that in this paper the authors make of this DAPI quantification a pillar for the whole research presented, it is not enough to be convincing. The authors should show first that DAPI quantification by fluorescence microscopy is an exact measure of the DNA content couple.
Moreover, they should couple DAPI quantification with another DNA staining such as propidium iodide which is commonly used to measure DNA content at least in cytofluorimetry. Nevertheless, this would not be an accurate DNA quantification but only an indication. Quantification of heterochromatin markers would be more informative, but this is something that it has been already widely observed previously.
-Results- First paragraph and figure 1- The authors should mention the criteria they used to define a nuclear bleb instead of a micronuclei. As said above, this is an essential point for a correct characterization and to support the authors' data throughout the whole manuscript. The image they show in figure 1, for example, can resemble a micronucleus.

© 2024. Published by The Company of Biologists under the terms of the Creative Commons Attribution License (https://creativecommons.org/licenses/by/4.0/).

“In wild type MEF cells the mean intensity measurements revealed significantly decreased DNA concentration in the nuclear bleb relative to body 0.55 ± 0.03 Figure 1, A and B)“ : It is not clear how the authors measure the intensity and how the ratio was performed. The areas of the ROIs considered are quite different and could explain the ratio below 1. The authors should be more specific and clarify in both the results and the methods section the criteria used for the quantification. Probably, it would be useful to show not only the ratio bleb/nucleus but also the total fluorescence (raw/integrated density) per area for both bleb and nucleus. The same applies to figure 1D and all the DNA quantifications throughout the text. The authors should also measure the DNA content in another normal cell line in humans, such as RPE-1, to corroborate their results.
Figure 1A: even though the authors indicated a nuclear blebbing in the WT sample as LaminB1 negative, it is still possible to see Lamin B1 staining though less intense than in the main nucleus. The authors should clarify this.
“Thus, chromatin decompaction results in similar decreased levels of DNA in the bleb compared to body as wild type, but a greater decrease in lamin B1 levels in the bleb compared to the body.” : could be this due to the fact that in one case we are looking to micronuclei and in the other to real blebs? The authors could comment on that. This is a doubt that emerges from most of the images of the nuclei presented in the manuscript in both MEFs, HCT116 and the cell lines from diseases.

Reviewer 3

Advance summary and potential significance to field

The submitted manuscript presents data indicating that a reduction in chromatin staining intensity is a more consistent indicator of nuclear blebs than a reduction in lamin b1 intensity. Nuclear blebs are important structural defects in the nucleus observed in both normal and pathological conditions. The authors demonstrate that DNA intensity is reduced in blebs across multiple cell types and following treatment with drugs that modify chromatin architecture while lamin B1 levels exhibit greater variability in blebs. While this study is useful, there are some inconsistencies in the results and great statistical rigor and a more measured description of the results would be advisable.

Comments for the author

1. Since the major thrust of the manuscript is that the variability of LB1 staining is greater than the variability in DAPI staining in blebs, it is critical that proper statistical test is used to quantify this difference. Statistical measures to test for equality in variance such as the Levene test have not been performed and should be carried out.
2. It would be useful to plot the intensities for most of the graphs as frequency distributions so as to give the reader a better idea of how the intensities vary and provide an idea of the proportion of cells with different bleb/body relative intensity values.
3. It would also be helpful to quantify bleb size and correlate it with either DNA or Lamin B1 intensity.
This might provide a more informative and mechanistic insight into why Lamin B1 intensity is more variable.
Previous reports have suggested that Lamin B1 might be excluded from smaller blebs.
4. The results that deal with correlation of the nuclear pore complex blebs are not obvious from the images provided and require quantification to support the authors assertion that there is a correlation between lamin B1 loss and NPC presence in nuclear blebs.
5. In Fig. 2D, the variation in the WT DNA bleb/body relative intensity appears quite different from the WT DNA bleb/body relative intensity in B and more similar to the Lamin B1 bleb/body relative intensity.
Quantification and statistical testing of the differences variances is critical for all these experiments.
6. The authors state that the live cell imaging recapitulates the results from the fixed cells but this not the case for the differences between untreated and VPA treated cells. In VPA treated cells, LB1 I bleb/body relative intensity is reduced when compared to WT (Fig. 1C) but they are not different in the live imaged cells (Fig. 2A and B).

© 2024. Published by The Company of Biologists under the terms of the Creative Commons Attribution License (https://creativecommons.org/licenses/by/4.0/).

7. On page 7 - “Oppositely, Lamin B1 levels varied drastically between the two different model cell lines (Figure 3, D and E).” – Please provide a statistical comparison between these two different model cell types.
8. Is the difference in the LB1 levels in the belbs related to the total LB1 levels in the cells, it would be useful to determine that.
9. On page 7 - “Lamin B1 was consistently absent in nuclear blebs compared to the nuclear body in both wild type (hTERT) and NGPS fibroblasts (Figure 3, A and D)” – Not supported by the data. There is a variation with numerous cells having LB1 in belbs, please provide a more accurate description in the text.
10. “Nuclear blebs are both hallmarks and a major cause of human diseases.” Please provide a reference to support that belbs are a cause of human disease.

First revision

Author response to reviewers' comments

Reviewer 1 Advance Summary and Potential Significance to Field:
In this manuscript Bunner et al. characterize DNA labeling density versus lamin B labeling on nuclear blebs arising in multiple cell lines and conditions, including in response to altered chromatin states, nuclear lamina organization, and cancer types, and find that decreased DNA density is a more robust indication of a nuclear bleb than loss of lamins or nuclear pore complexes (NPCs).
This is a necessary study that seeks to clarify a persistent issue in the field with identifying and classifying nuclear morphology changes and provides a new tool for researchers to accurately quantify nuclear blebs. The authors take several approaches to validate their approach, including assessing protein and DNA intensity in fixed and live cells and by partial wave spectroscopy. Each approach shows a consistent decrease in DNA density in nuclear blebs across multiple conditions. This study could be of significant interest to researchers studying nuclear envelope structure, nuclear mechanics, and cancer cell biology. A major limitation is that it does not clarify how nuclear blebs are distinguished from a major contaminating object class - micronuclei - and it is not clear that the widefield imaging system used has the power to do so.
Author: We appreciate the Reviewers’ clear comments on how to make the manuscript better. While we used clear parameters for separating nuclear blebs from micronuclei, that was not sufficiently discussed in the original manuscript. The other reviewers echo this need. In the revised manuscript we:
1) Clarify that nuclear blebs form during interphase, and micronuclei form during mitotic error.
2) Provide new data via new additional main Figure 1 and Supplemental Figure 1 that provides a clear protocol for separating nuclear blebs from micronuclei.
3) We also provide new data to address why lamin B levels in the nuclear bleb are variable and change significantly between cell types and perturbations via new main Figure 6.

Reviewer 1 Comments for the Author:
I would support the publication of this manuscript if the following points can be addressed:

Major Points

Reviewer 1- 1. Both micronuclei and nuclear blebs can appear as round DNA positive and often lamin positive objects abutting the nucleus. Although some images of lamin B1 positive blebs show a clear continuation between the DNA or lamin in the nucleus in the bleb (e.g. Fig. 1A, WT, bleb LB1+), as expected, in many cases such continuity is not visible or noticeably absent in this class of objects (e.g. Fig. 1A LA KD bleb LB1+, left; Fig. 1D WT; Fig. 2A; Fig. 3A LNCaP). In contrast, continuity of the DNA is easily visible in all of the LB1- examples. In addition, the live-cell imaging example of a lamin B1 positive bleb forming (Fig. 2C), which would be very conclusive that blebbing is a major mechanism of lamin B1/DNA positive object

© 2024. Published by The Company of Biologists under the terms of the Creative Commons Attribution License (https://creativecommons.org/licenses/by/4.0/).

formation, shows an extruded object with much lower levels of lamin B1 than in many of the example images, consistent with multiple classes of objects being included in the lamin B1+ category.

A critical part of the bleb definition is that the DNA and nucleoplasm are continuous with the nucleus. With the widefield imaging set up available, this could mean identifying focal planes for all the images where the DAPI appears to change intensity gradually between the nucleus and bleb, which could be visualized in a line scan. Ideally the authors would repeat these analyses on a confocal or deconvolution system with a nuclear envelope marker that would allow them to show that the blebs have a continuous rim of lamin B1 with the nucleus. In addition, the authors should induce large micronuclei in these cells, for instance through nocodazole arrest or inhibition of the mitotic checkpoint, to determine how frequently large micronuclei have decreased DNA intensity and normal lamin B1 labeling in their cell lines. Although recent work suggests that micronuclear DNA is compact and that lamin levels can be very low, there may be significant differences across cell lines and conditions.

Author response: The reviewer overall is asking for us to clarify our procedure for determining nuclear blebs vs. micronuclei. In the revised manuscript we have now provided a new figure (Figure 1) that provides data for separation of nuclear bleb vs. micronuclei. Our protocol for deciding the nuclear bleb is a contiguous via line scan of DNA reporter (SiR-DNA, Hoechst, DAPI) while micronuclei show clear separation from the nucleus (Figure 1, A and B).

Furthermore, we have provided new data time lapse imaging live cells with NLS-GFP and SiR-DNA to show that nuclear blebs are connected to the nuclear body moving in tandem, especially rotating together (Figure 1C), while micronuclei show independent and separated movement (Figure 1D). We find that MEF nuclei labeled with SiR-DNA recapitulates that DNA density in nuclear blebs is decreased on average 33% relative to the nuclear body with a tight range of 0.8 – 0.45 relative to the nuclear body set to 1.0 (Figure 1E). Oppositely, micronuclei show 50/50 split of either increased or decreased DNA relative to the nucleus (< 0.9 >) with large variability and average 0.96 which is significantly higher than nuclear blebs (t-test $p < 0.05$, Figure 1E). Through live cell tracking we find that a micronucleus, which occurs infrequently in our cells, touches the nucleus on average ~9% over a 3-hour period imaged at 2-minute intervals (Figure 1F). Thus, in a static image, our ability to determine a micronucleus is not a nuclear bleb has an 9% failure rate. Furthermore, in half of those events (4.5%) the DNA density of the micronucleus is at or above the level of the nucleus, providing a clear secondary metric to rule out possible micronuclei being confused for a bleb.

In the revised manuscript we provide new measurements of micronuclei relative to the nucleus for HCT 116 and all prostate cancer cell lines (LNCaP, PC3, and DU145). Similar to MEF micronuclei, measurements of relative DNA intensity of micronuclei to the nucleus in these cell lines (expect PC3) is significantly higher than nuclear blebs (t-test $p <0.05$, Supplemental Figure 1A). Thus, micronuclei on average show greater DNA concentration than nuclear blebs with some even showing similar or higher DNA concentration compared to the nucleus.

Oppositely, MEF, HCT116, LNCaP, PC3, DU145, hTERT WT and NGPS, and newly added HT1080 and RPE-1 in Supplemental Figure 1 all show consistently decreased levels of DNA concentration of which >95% are < 0.80 relative to the main nuclear body.

This new data provides a different approach that is strongly supported by the original manuscripts live cell imaging of HCT116 nuclei in Figure 3. This data showed that nuclear blebs forming in interphase could have variable lamin B1 levels in the nuclear bleb while DNA intensity was consistently decreased for DNA intensity. In those movies, those nuclear blebs move with the nuclear body providing verification.

Finally, this reviewer and the others are attempting to explain why lamin B1 levels are variable and why they change from cell line to cell line and perturbation to perturbation. In the absence of data, reviewers are concerned these variations are due to micronuclei being confused for nuclear blebs. We appreciate this feedback which has driven us to provide new data supporting a clear mechanism for why lamin B1 levels vary in nuclear blebs. In the revised manuscript we provide new data in a new Figure 6 showing that lamin B1 levels in the nuclear bleb are dependent on nuclear rupture. Specifically, we time lapse imaged MEF NLS-GFP cells over 3 hours at 3-minute intervals to measure nuclear blebbing via nuclear shape and rupture via NLS-GFP spilling into the cytoplasm. This was done on a gridded dish, so that immediately after the time lapse, we could conduct immunofluorescence on the same cells to measure DNA and lamin B1 in the nuclear bleb. Nuclear blebs that ruptured within 30 minutes of the end to the time lapse had a significantly decreased lamin B1 level relative to nuclear blebs that were stable (did not rupture). Thus, we provide a clear mechanism – nuclear rupture or stability of the bleb –

© 2024. Published by The Company of Biologists under the terms of the Creative Commons Attribution License (https://creativecommons.org/licenses/by/4.0/).

drives lamin B1 variability and possible changes between different cell lines and perturbations. Finally, we clarify that lamin B1 positive blebs are consider > 0.9 relative to the body while lamin B1 negative is < 0.9. We agree with the reviewer that lamin B1 signal is a spectrum and thus we use these words to bin this spectrum into a binary output for ease of discussing the data.

Reviewer 1-2. The NPC antibody used in figure 1A has too much background to determine its localization at the nucleus periphery. Although 414 is a standard NPC marker, Nup133 and Nup153 are also common enumeration labels that frequently give a cleaner signal than 414.
Author response: In response to the reviewer's comment (echoed by other reviewers), we have removed the images of the NPC marker and text referencing it in the revised manuscript. Originally, it was our goal to show interesting qualitative results. However, we agree that the staining quality can be better, and quantification can be done, though these experiments are beyond the scope of this manuscript. Instead, we now focus only on the quantitative data of decreased DNA concentration in the bleb compared the highly variable lamin B1 staining. This focus of the manuscript has led to the inclusion of two new main figures and one supplemental figure worth of data to the manuscript to support our main conclusion (Figure 1, Figure 6, and Supplemental Figure 1).

Minor point

Reviewer 1- 3. The resolution of Fig. 4 is significantly lower than the rest making it harder to read and see what's going on in the nucleus renderings.
Author response: We appreciate the reviewer's feedback and have reproduce this figure to increase resolution which is now Figure 5 in the revised manuscript.
*****
Reviewer 2 Advance Summary and Potential Significance to Field:
This is an interesting work addressing the question of the composition of the nuclear blebs, with the goal of defining the correct marker of a nuclear bleb. Though this is an important point in the biology of blebs and would be useful for the scientific community in the field, there are several issues that weaken authors' conclusions.
We thank the reviewer for stating the work is "interesting" and that the work "would be useful for the scientific community". Below we address each of the reviewer's points to improve the paper for publication.

Reviewer 2 Comments for the Author:
Below my specific comments that the authors could address to improve their manuscript in a revised version.

Reviewer 2- 1. One of the main problems with the presented results is that it is often unclear if the authors are considering real nuclear blebs or micronuclei that stay close to the surface of the main nucleus. This is an important point, because micronuclei could have lamina, and this could be the reason why the authors observe variability in all the studied cell lines in the presence/absence of lamin B1 in the supposed nuclear blebs. The authors should discuss this. Another problem is the methods used to quantify DNA as the measurement of the fluorescence intensity of DAPI, Hoechst and siRDNA is not commonly considered reliable. Both issues undermine the validity of authors' conclusions.
Author response: The reviewer raises two points that we address:
1) How to differentiate between a nuclear bleb herniating from the nuclear body and micronuclei that are not attached but just close to the nuclear body?
Newly added data in the revised manuscript via newly added Figure 1:

a. **Line scans** - We have provided new data showing we use line scans to determine nuclear blebs which are protrusion of the main nuclear body from micronuclei that are separate from the nucleus (Figure 1, A and B).
b. **Time lapse imaging** – We provide new data tracking nuclear blebs and micronuclei over time at 2-minute intervals for 3 hours in MEF cells. This timelapse data provides the definitive ability to determine a nuclear bleb, which moves with the nucleus

© 2024. Published by The Company of Biologists under the terms of the Creative Commons Attribution License (https://creativecommons.org/licenses/by/4.0/).

because it is a part of it, and a micro nucleus which is a separate item. (Figure 1, C and D)

c. **Time lapse verified nuclear bleb and micronuclei SiR-DNA relative intensity** - We find that nuclear blebs are consistently between 20-50% decreased density relative to the nuclear bleb (0.8-0.5 relative to nuclear body 1.0) with an average of
0.68. This average is similar to that reported consistently across many types of cells. Differently, micronuclei vary drastically with about half showing at or higher levels of DNA density than the nucleus and an average of 0.96 relative to nuclear body at 1.0. (Figure 1E). This also provides a secondary metric for separating micronuclei from nuclear blebs.
d. Our new time lapse data show that micronuclei are rarely (9%) close enough to nuclei to be confused as nuclear blebs via line scan, suggesting we have a low rate of confusing a micronucleus to a bleb.
e. We now provide new data via Supplemental Figure 1 measuring the relative intensity of micronuclei to the nucleus in HCT116 and all prostate cancer cells lines (LNCaP, PC3, and DU145). Similar to MEF micronuclei, these micronuclei present higher relative DNA concentrations (t-test, $p < 0.05$) which can be at or above nucleus concentration, except for PC-3 for which we only found n =4 micronuclei.

Other newly added data addressing lamin B1 variability: Again, the reviewers provide hypotheses for lamin B1 variability because we did not address this in the original manuscript. In the revised manuscript we provide new data in the newly added Figure 6 showing the lamin B1 level in the nuclear bleb is dependent on recent rupture of the nuclear bleb or lack thereof, which we call stable blebs. Using a gridded dish allows us to conduct time lapse imaging and immunofluorescence on the same cells and their nuclei. The 3-hour time lapse of NLS-GFP allows for accurate separation of nuclear blebs from micronuclei AND tracking nuclear rupture. The following immunofluorescence of the same cells allows for us to determine the levels of DNA and lamin B1 in the nuclear bleb. A nuclear bleb that ruptured during the time lapse had significantly decreased lamin B1 levels relative to the nuclear body 0.49 ± 0.04 compared to stable (non-rupture) nuclear blebs that showed higher levels of lamin B1 at 0.77 ± 0.04. As we show throughout the manuscript, decreased DNA concentration in the nuclear bleb was consistent and similar in both ruptured or stable blebs.

Original manuscript - This point was addressed in the original manuscript in Figure 3, where live cell imaging of nuclear blebs forming can have varying levels of lamin B1. Micronuclei form during mitotic division. Oppositely, nuclear blebs are herniations that occur in interphase. We imaged normally shaped interphase nuclei form a bleb during the time lapse which could be lamin B1 positive (similar to nuclear body) or negative (significantly decreased).

2) How reliable are DNA fluorescence markers DAPI, Hoechst, SiR-DNA?

This criticism is directly addressed in the original manuscript by PWS measurements in Figure 5. PWS measures chromatin density through scattering of light which measures a similar significant decrease in the DNA density/concentration in the nuclear bleb compared to the body. Overall, we do not agree with the reviewer's statement the DAPI, Hoechst, and SiR-DNA "is not considered reliable". We and many others have previously published that increased DNA content changes during the cell cycle by these DNA markers, as the total fluorescence intensity doubles from G1 to G2. <u>We have included new data</u> in the revised manuscript showing that these DNA dyes provide quantitative data on DNA amount. HT1080 FUCCI cells that provide fluorescence reporters for G1 cells which have diploid genome vs late S/G2 cells that have tetraploid genome. In short, Hoechst measured DNA content nearly doubles from the G1 to G2, as expected (Supplemental Figure 1 B-E). Furthermore, we are measuring the difference in DNA concentration inside the same nucleus (bleb vs. body) providing a rigorous control. The DNA concentration in the nuclear bleb is less than that of the nucleus from which it herniated.
This point is addressed further in reviewer point #5, which shows Hoechst can be used to measure quantitative DNA content via published reports, which we now cite in the revised manuscript.

Specific points:

Reviewer 2-2. Abstract- " Chromatin has been reported to be decreased or decompacted in nuclear blebs": the authors are probably referring to heterochromatin and not generally chromatin that is decreased, they should rephrase the sentence.

Author response: The reviewer raises a valid point. Thus, we have revised the text to remove

© 2024. Published by The Company of Biologists under the terms of the Creative Commons Attribution License (https://creativecommons.org/licenses/by/4.0/).

the word decreased.

Reviewer 2- 3. Introduction- “Studies over the last 20 years have deduced the nuclear components that are essential for maintaining normal ellipsoidal nuclear shape are chromatin…”: the authors should be more specific and mention that the epigenetic modifications play an essential role in this regulating chromatin compaction degree as discussed in recent reviews.
Author response: We have made the suggested revision.

Reviewer 2-4. Introduction- The authors should mention the difference between nuclear blebs and micronuclei, which is an important piece of the puzzle they want to address in their manuscript. A distinction between the two phenotypes could be indeed important to unravel the question of the real composition of the nuclear blebs as reported previously (Utani et al. PlosONE 2010, 2011).
Author response: We appreciate the reviewer’s comment and have added a text to the revised manuscript’s introduction to address this point.

Reviewer 2-5. Results- “To determine the composition of nuclear blebs, we applied immunofluorescence imaging to label nuclei for DNA (DAPI) …”- DAPI staining is not usually considered suitable for quantitative measurements of DNA, among other things it only binds A-T rich sequences, as such the results the authors are showing are not conclusive nor indicative. However, it is true that there is in literature an indication that reducing DAPI concentration it could be used for quantification by cytofluorimetry, but the authors did not mention the concentration of DAPI they used, nor the parameters used at the microscope to acquire the DAPI images, instead it would seem that they simply used a common mounting media containing DAPI as reported in the methods section. In previous publications the authors used this method (DAPI/Hoechst fluorescence intensity) to quantify DNA, however, considering that in this paper the authors make of this DAPI quantification a pillar for the whole research presented, it is not enough to be convincing. The authors should show first that DAPI quantification by fluorescence microscopy is an exact measure of the DNA content couple.
Moreover, they should couple DAPI quantification with another DNA staining such as propidium iodide which is commonly used to measure DNA content at least in cytofluorimetry. Nevertheless, this would not be an accurate DNA quantification but only an indication. Quantification of heterochromatin markers would be more informative, but this is something that it has been already widely observed previously.

Author response:
A) As mentioned in Reviewer point 1 above, this criticism has been largely addressed in the original manuscript in which we use PWS measurements of density to determine that the nuclear bleb contains less DNA concentration (Figure 5). Partial Wave Spectroscopy uses light scatter to determine density and has been shown to measure changes in chromatin nano- architecture (Almassalha, et al 2016 PNAS). Thus, two different techniques, DNA dye imaging and PWS, measure decreased DNA concentration/density in the nuclear bleb relative to the nuclear body.
B1) The reviewer’s comments are contradicted by published data. These DNA dyes can provide quantitative data on relative DNA amount as shown in: Assaying cell cycle status using flow cytometry by Kang Ho Kim and Joel M. Sederstrom Curr Protoc Mol Biol. PMCID: PMC4516267; PMID: 26131851. The data from this paper (provided below) shows these DNA dyes are just as good as the reviewers preferred propidium iodide.
Figure provided for reviewer has been removed. It showed in Figure 1 from Sederstrom and Kim (2015)Assaying cell cycle status using flow cytometry Curr Protoc Mol Biol. (doi:10.1002/0471142727.mb2806s111)

B2) We now include data showing that FUCCI labeled nuclei to determine G1 (Cdt1) and late S/G2 (Geminin) shows a twofold increase on total DAPI signal. (Supplemental Figure 1 B-E)
B3) Furthermore, this labeling is of DNA in the same nucleus differentiating nuclear body vs. bleb. Thus, any bias for staining would be held constant between the two, body and bleb.
C) The statement that “Quantification heterochromatin markers would be more informative…” does not fall in the scope of the paper. We are investigating DNA concentration, not chromatin

© 2024. Published by The Company of Biologists under the terms of the Creative Commons Attribution License (https://creativecommons.org/licenses/by/4.0/).

histone modification state. Furthermore, heterochromatin is composed of many specific histone modifications (H3K9me3, H3Kme2, H3K27me3 to name a few) and thus would require an entire other manuscript to address this suggested approach making it clearly beyond the scope of the current manuscript.

Reviewer 2-6. Results- First paragraph and figure 1- The authors should mention the criteria they used to define a nuclear bleb instead of a micronuclei. As said above, this is an essential point for a correct characterization and to support the authors' data throughout the whole manuscript. The image they show in figure 1, for example, can resemble a micronucleus.
Author response: We agree with the reviewer's point and have provided new data and text revisions to address this important point. As mentioned previously in the response (see point #1), we have included new data in the revised manuscript via a newly added Figure 1 defining the protocol used to differentiate a nuclear bleb from a micronucleus. This includes line scan, quantification of DNA concentration relative to nuclear body, time lapse data verification, and providing a failure rate of 9% in which a micronucleus might be confused for a nuclear bleb in a static image. Furthermore, micronuclei show higher DNA concentrations in which 50% are > 0.9 relative to the nuclear body providing a secondary metric to distinguish blebs (tracked via timelapse, 100% < 0.9) from micronuclei.

Reviewer 2-7. "In wild type MEF cells the mean intensity measurements revealed significantly decreased DNA concentration in the nuclear bleb relative to body 0.55 ± 0.03 Figure 1, A and B)" : It is not clear how the authors measure the intensity and how the ratio was performed. The areas of the ROIs considered are quite different and could explain the ratio below 1. The authors should be more specific and clarify in both the results and the methods section the criteria used for the quantification. Probably, it would be useful to show not only the ratio bleb/nucleus but also the total fluorescence (raw/integrated density) per area for both bleb and nucleus. The same applies to figure 1D and all the DNA quantifications throughout the text. The authors should also measure the DNA content in another normal cell line in humans, such as RPE-1, to corroborate their results.
Author response: We have revised the text of the Results and Methods section as requested. We specifically use average intensity because this normalizes for size of the object/ROI. Between small and larger objects, the integrated intensity changes drastically largely to change in size but not concentration.
We have provided new measurements of more cell lines as requested by the reviewer. We now show that both HT1080 and RPE-1 nuclear blebs show a similar decreased DNA concentration via decreased average Hoechst fluorescence intensity relative to the nuclear body. (Supplemental Figure 1). We would like to point out that our revised manuscript now shows that mouse MEF cells and Human cell lines HCT116, fibroblasts hTERT, NGPS, LNCaP, PC3, DU145, HT1080, and RPE-1 all consistently show a significant decrease in DNA concentration in the nuclear bleb compared to the nuclear body.

Reviewer 2-8. Figure 1A: even though the authors indicated a nuclear blebbing in the WT sample as LaminB1 negative, it is still possible to see Lamin B1 staining though less intense than in the main nucleus. The authors should clarify this.
Author response: The reviewer is correct that lamin B1 "negative" blebs still have lamin B1 staining but just less of it. We have revised the figure legend to clarify that lamin B1 positive denotes similar levels as nuclear body while negative denotes lower levels. Specifically, we provide a threshold of 0.9 relative intensity to determine positive vs. negative.

Reviewer 2-9. "Thus, chromatin decompaction results in similar decreased levels of DNA in the bleb compared to body as wild type, but a greater decrease in lamin B1 levels in the bleb compared to the body." : could be this due to the fact that in one case we are looking to micronuclei and in the other to real blebs? The authors could comment on that. This is a doubt that emerges from most of the images of the nuclei presented in the manuscript in both MEFs, HCT116 and the cell lines from diseases.
Author response: Our new data in the revised manuscript addresses this point via two newly added figures: Figure 1 and Figure 6. In Figure 1, time lapse imaging shows that micronuclei touch the nucleus rarely at 9%. Furthermore, Figure 1 data shows that 50% of micronuclei have a DNA concentration similar to or greater than the nuclear body. Taken together confusing a micronucleus for a bleb is rare and when it does occur it can be obvious due to increased DNA

© 2024. Published by The Company of Biologists under the terms of the Creative Commons Attribution License (https://creativecommons.org/licenses/by/4.0/).

concentration, which does not occur in nuclear blebs confirmed via time lapse imaging.
Again, this reviewer, like the other reviewers, is providing a hypothesis for why lamin B1 levels vary. We fully understand not addressing this point left room for the reviewers and future readers alike to question the mechanism. In the revised manuscript, we provide newly added data in Figure 6 that provides a mechanism for lamin B1 level variations. Using a gridded dish, we provide data from time lapse imaging NLS-GFP to track nuclear shape and rupture into immunofluorescence to measure DNA and lamin B1 levels of the nuclear bleb. Our data show that recently ruptured nuclear blebs have low lamin B1 (0.49) while stable/non-rupture nuclear blebs have significantly higher lamin B1 levels (0.77). Thus, the data refute the hypothesis that variation in lamin B1 is due to confusion of micronuclei as blebs. Instead, the data support that nuclear bleb rupture or stability determines lamin B1 level variation.

*****

Reviewer 3 Advance Summary and Potential Significance to Field:

The submitted manuscript presents data indicating that a reduction in chromatin staining intensity is a more consistent indicator of nuclear blebs than a reduction in lamin b1 intensity. Nuclear blebs are important structural defects in the nucleus observed in both normal and pathological conditions. The authors demonstrate that DNA intensity is reduced in blebs across multiple cell types and following treatment with drugs that modify chromatin architecture while lamin B1 levels exhibit greater variability in blebs. While this study is useful, there are some inconsistencies in the results and great statistical rigor and a more measured description of the results would be advisable.

Reviewer 3 Comments for the Author:

Reviewer 3-1.Since the major thrust of the manuscript is that the variability of LB1 staining is greater than the variability in DAPI staining in blebs, it is critical that proper statistical test is used to quantify this difference. Statistical measures to test for equality in variance such as the Levene test have not been performed and should be carried out.

Author response: We would like to thank the reviewer for this feedback which has strengthened the revised manuscript.

- We have revised the manuscript to clarify that our main point is that lamin B1 levels change significantly between cell lines and perturbations.
- In the revised manuscript we now use the Levene test to provide statistical support for increased variance of lamin B1 compared to DNA in nuclear blebs. In all areas where we stated increased variability, we now have the accompanying Levene test to support this statement.

Reviewer 3-2.It would be useful to plot the intensities for most of the graphs as frequency distributions so as to give the reader a better idea of how the intensities vary and provide an idea of the proportion of cells with different bleb/body relative intensity values.

Author response: In the original and revised manuscripts, we provide each data point in the graphs so the reader can see the distribution and range of data. We largely discuss the data of bleb intensities for DNA and lamin B in terms of relative to the body using the cutoff of <0.9> for decreased vs. similar. We have also now included this reviewer's Leven test for variability, which we believe is clear in the graphs showing each nucleus measured. Re-graphing all of our data into frequency distributions does not support a major conclusion. Thus, we will maintain showing the raw data of individual measures in our graphs.

Reviewer 3-3.It would also be helpful to quantify bleb size and correlate it with either DNA or Lamin B1 intensity. This might provide a more informative and mechanistic insight into why Lamin B1 intensity is more variable. Previous reports have suggested that Lamin B1 might be excluded from smaller blebs.

Author response: Similar to other reviewers, hypotheses were put forward to understand lamin B1 variation. To address this important point in the revised manuscript we provide newly added data in Figure 6 that reveals lamin B1 levels are dependent on nuclear bleb rupture or stability (non-rupture). For this data we performed analysis of nuclear bleb size and found no statistically significant change in nuclear bleb size (rupture 22 ± 3 um^2 vs. stable blebs 18 ± 4 um^2, p = 0.32 t-test).

Reviewer 3-4.The results that deal with correlation of the nuclear pore complex blebs are not obvious from the images provided and require quantification to support the authors assertion that there is a correlation between lamin B1 loss and NPC presence in nuclear blebs.

Author response: We have removed the qualitative images of NPC immunofluorescence from the

© 2024. Published by The Company of Biologists under the terms of the Creative Commons Attribution License (https://creativecommons.org/licenses/by/4.0/).

revised manuscript, which was requested by many reviewers. We agree that optimizing the immunofluorescence and quantifying the data would be most ideal. However, our initial inclusion was to show interesting qualitative observations. Providing NPC data is currently beyond the scope of this manuscript focused on comparing DNA and lamin B1 levels in nuclear blebs. To this point we have added two new main Figures 1 and 6 as well as one Supplemental Figure to support the main scope of this work.

Reviewer 3-5.In Fig. 2D, the variation in the WT DNA bleb/body relative intensity appears quite different from the WT DNA bleb/body relative intensity in B and more similar to the Lamin B1 bleb/body relative intensity. Quantification and statistical testing of the differences variances is critical for all these experiments.

Author response: We believe the reviewer meant original Figure 1 data that is now Figure 2 in the revised manuscript. WT DNA bleb/body intensity in Figure 2B and 2D is statistically similar while WT DNA vs LB1 is significantly different in both Figure 2B/C and 2D. We have now included these statistical measures in the respective results text which again is highlighted in red text.

Reviewer 3-6.The authors state that the live cell imaging recapitulates the results from the fixed cells but this not the case for the differences between untreated and VPA treated cells. In VPA treated cells, LB1 I bleb/body relative intensity is reduced when compared to WT (Fig. 1C) but they are not different in the live imaged cells (Fig. 2A and B).

Author response: We thank the reviewer for pointing out that our language needs to be clarified. In the revised manuscript we clarify this point with a new added sentence “Interestingly, while MEF cells VPA-treated nuclei show a decrease in lamin B1 bleb levels compared to wild type (**Figure 2B**), this significant decrease of lamin B1 upon VPA does not occur in HCT116 (**Figure 3**).”

Reviewer 3-7.On page 7 - “Oppositely, Lamin B1 levels varied drastically between the two different model cell lines (Figure 3, D and E).” - Please provide a statistical comparison between these two different model cell types.

Author response: In the revised manuscript we now include the significant p value comparing hTERT human fibroblasts with LNCaP early prostate cancer cell lines with data shown in Figure 4.

Reviewer 3-8.Is the difference in the LB1 levels in the belbs related to the total LB1 levels in the cells, it would be useful to determine that.

Author response: In the revised manuscript, we provide newly added Figure 6 data showing that variation in lamin B1 levels is due to the occurrence or absence of nuclear bleb rupture. In this same data lamin B1 levels in the nuclear body were similar for ruptured vs stable (2155 ± 110 vs 2070 ± 63 arbitrary units, t-test $p = 0.71$).

Reviewer 3- 9.On page 7 - “Lamin B1 was consistently absent in nuclear blebs compared to the nuclear body in both wild type (hTERT) and NGPS fibroblasts (Figure 3, A and D)” - Not supported by the data. There is a variation with numerous cells having LB1 in belbs, please provide a more accurate description in the text.

Author response: We agree with the reviewer that the text needs to be clarified. We have revised the manuscript to state that lamin B1 was drastically decreased in the majority of nuclear blebs.

Reviewer 3-10.“Nuclear blebs are both hallmarks and a major cause of human diseases.” Please provide a reference to support that belbs are a cause of human disease.

Author response: We have revised the text to state that “nuclear blebs are both hallmarks and a contributor to cellular dysfunction in human diseases”.

Second decision letter

MS ID#: JOCES/2024/262082

MS TITLE: Decreased DNA density is a better indicator of a nuclear bleb than lamin B loss

AUTHORS: Samantha Bunner, Kelsey Prince, Emily M Pujadas Liwag, Nebiyat Eskndir, Karan Srikrishna, Pedro Pellegrino, Julianna Aiello, Antonia Amonu McCarthy, Colman Lawlor, Shrushti Jagtap, Anna Kuklinski, Emma Eastin, Griffin Yas, Nathan LaPointe, Olivia Jackson, Imuetiyan Eweka, Isabelle Schramm von Blucher, Jillian Hardy, Jason Chen, Schuyler Figueroa, Vadim

© 2024. Published by The Company of Biologists under the terms of the Creative Commons Attribution License (https://creativecommons.org/licenses/by/4.0/).

Backman, Anne Janssen, Mary Packard, Katherine Dorfman, Luay Almassalha, Michael Seifu Bahiru, and Andrew D Stephens

We have now reached a decision on the above manuscript.

To see the reviewers' reports and a copy of this decision letter, please go to: https://submit-jcs.biologists.org and click on the 'Manuscripts with Decisions' queue in the Author Area. (Corresponding author only has access to reviews.)

As you will see, the reviewers gave favourable reports but raised some critical points that will require amendments to your manuscript. I hope that you will be able to carry these out because I would like to be able to accept your paper, depending on further comments from reviewers.

Please ensure that you clearly highlight all changes made in the revised manuscript. Please avoid using 'Tracked changes' in Word files as these are lost in PDF conversion.

I should be grateful if you would also provide a point-by-point response detailing how you have dealt with the points raised by the reviewers in the 'Response to Reviewers' box. Please attend to all of the reviewers' comments. If you do not agree with any of their criticisms or suggestions please explain clearly why this is so.

Reviewer 1

Advance summary and potential significance to field

In this manuscript Biswas et al. characterize DNA labeling density and lamin B labeling on nuclear blebs arising in multiple cell lines and conditions including in response to altered chromatin states, nuclear lamina organization and cancer types, and find that decreased DNA density is a robust indication of a nuclear bleb. This is a necessary study that seeks to clarify a persistent issue in the field with identifying and classifying nuclear morphology changes and provides a new tool for researchers to accurately quantify nuclear blebs.

Comments for the author

The authors have added new data and information to address the reviewers' major concern that micronuclei were being erroneously analyzed as nuclear blebs.
Unfortunately, their DNA line scan approach does not resolve the issue for the fixed cell images. Almost all of the LB1+ blebs in fixed cell images are consistent with micronuclei as the nuclear lamina of the blebs appears as a complete circle, distinct from the nucleus. There are strong examples of LB1+ blebs from fixed cells in Fig. 2A (WT MEF, left) and Fig. 4A (DU145), demonstrating that blebs can be distinguished from micronuclei with their imaging parameters.

The current data strongly suggest that decreased DNA concentration is a hallmark of nuclear blebs, but the conclusions about LB1 variability on blebs between conditions and disease models are still not adequately supported. The alternative hypothesis, that these conditions alter micronucleation frequency and/or appearance and that these are the major source of high LB1 levels in "blebs", is equally likely based on the representative images. The most compelling evidence that decreased DNA density is a consistent maker of blebs is the HCT116 GFP-B1 live cell imaging analysis where multiple criteria are used to identify blebs. However, in these data LB1 is uniformly depleted in blebs (Fig. 3B), unlike in the analysis of fixed cells (Fig. 3A), suggesting that the fixed cell criteria are including non-blebs in the analysis.

The finding that DNA density in blebs is consistently reduced is strong enough to merit publication of this paper and I am concerned this will get lost in disagreements about what is being analyzed. Therefore, I strongly recommend the authors reanalyze their fixed images using the more conservative criterium of NL connectedness to distinguish blebs from micronuclei and report the results.

Minor comments:
1. Decreasing the point opacity on the dot plots would improve readability.
2. Fig. 2B – add stats to graph.

© 2024. Published by The Company of Biologists under the terms of the Creative Commons Attribution License (https://creativecommons.org/licenses/by/4.0/).

3. Fig. S1A – these data are hard to interpret without the nuclear bleb intensity values on the same graph, especially for the prostate cancer cell lines.
4. The description of the causal link between LB1 in the bleb and bleb rupture is inconsistent between the results and the discussion. The results section implies that rupture causes a loss of LB1 whereas the discussion implies that LB1- blebs have an increased likelihood of rupture. The second statement is strongly supported by the literature, whereas the first one requires additional experiments to validate. The results section should therefore be modified to match the discussion section.

Reviewer 2

Advance summary and potential significance to field

This is a second round of review of an interesting work addressing the question of the composition of the nuclear blebs, an important point in cell biology.

Comments for the author

I would like to thank the authors for considering my concerns and providing a revised version of their manuscript according to the Reviewers' comments.
The authors satisfactorily replied to most of my comments and I think that the manuscript has much improved, however I still have some concerns indicated below.

Major comments:
1) About the point of "How reliable are DNA fluorescence markers DAPI, Hoechst SiR-DNA" the authors added some experiments quantifying DAPI fluorescence relative to cell cycle phases markers (geminin/cdt1) and replied that "We and many others have previously published that increased DNA content changes during the cell cycle by these DNA markers, as the total fluorescence intensity doubles from G1 to G2. We have included new data in the revised manuscript showing that these DNA dyes provide quantitative data on DNA amount. HT1080 FUCCI cells that provide fluorescence reporters for G1 cells which have diploid genome vs late S/G2 cells that have tetraploid genome. In short, Hoechst measured DNA content nearly doubles from the G1 to G2, as expected (Supplemental Figure 1 B-E)." It is true that if we consider G1 vs lateS-G2 DNA nuclear content can be estimated by DAPI signal. This is straightforward since the content is doubled in these two different moments of the cell cycle. However, the authors here are not quantifying DNA of the cells that are going through cell division. And even for cell cycle analysis by flow cytometry DAPI is not frequently used because it preferentially stains AT-rich DNA thus underestimating DNA content. The authors here, would like to use DAPI (or siRDNA or HOECHST) fluorescence in IF to measure the different DNA content between nuclear body and bleb within the same nucleus in whatsoever cell cycle phase. DAPI staining, as it is clearly noticed also in the manuscript figures, generally does not stain the nucleus in a homogeneous way, it depends on the level of DNA condensation. This is not taken into account in the manuscript nor in the authors' reply and I am afraid that this could be an essential issue in the context. Considering the delicacy of the matter and the importance of this data, I would rather use the term DNA density or better DAPI signal instead of DNA amount/concentration/quantity/content.
Accordingly, the authors should rephrase this part of the manuscript and the following claims based on this type of quantification and in the abstract.
2) Related to 1) the authors did not provide a comparison with another more proper DNA staining such as Propidium Iodide as suggested in the first review.
The authors replied that DAPI staining is as quantitative as PI, however they show published paper about flow cytometry analysis which is way different from microscope images analysis. Comparing with staining with FUCCI system only tells the phase of the cell cycle and not the exact DNA content in the analysed conditions.

Minor Comment:
-Authors' reply:" C) The statement that "Quantification heterochromatin markers would be more informative…" does not fall in the scope of the paper. We are investigating DNA concentration, not chromatin histone modification state.

© 2024. Published by The Company of Biologists under the terms of the Creative Commons Attribution License (https://creativecommons.org/licenses/by/4.0/).

Furthermore, heterochromatin is composed of many specific histone modifications (H3K9me3, H3Kme2, H3K27me3 to name a few) and thus would require an entire other manuscript to address this suggested approach making it clearly beyond the scope of the current manuscript."
I understand that checking the epigenetic markers could have been dispersive and partially repetitive due to already published papers on the matter, however, the authors only provide DAPI staining to quantify DNA concentration and, again DAPI staining quantification by IF is not a fine methodology to quantify DNA.

Reviewer 3

Advance summary and potential significance to field

The submitted manuscript presents data indicating that a reduction in chromatin staining intensity is a more consistent indicator of nuclear blebs than a reduction in lamin b1 intensity. This will be a useful criterion in the identification of blebs and can also provide mechanistic insight into their organization and function.

Comments for the author

The authors have addressed all my concerns and have now carried out all the appropriate statistical and editorial modification that I suggested.

Second revision

Author response to reviewers' comments

Reviewer 1 Advance Summary and Potential Significance to Field:
In this manuscript Biswas et al. characterize DNA labeling density and lamin B labeling on nuclear blebs arising in multiple cell lines and conditions, including in response to altered chromatin states, nuclear lamina organization, and cancer types, and find that decreased DNA density is a robust indication of a nuclear bleb. This is a necessary study that seeks to clarify a persistent issue in the field with identifying and classifying nuclear morphology changes and provides a new tool for researchers to accurately quantify nuclear blebs.
Reviewer 1 Comments for the Author:
The authors have added new data and information to address the reviewers' major concern that micronuclei were being erroneously analyzed as nuclear blebs. Unfortunately, their DNA line scan approach does not resolve the issue for the fixed cell images. Almost all of the LB1+ blebs in fixed cell images are consistent with micronuclei as the nuclear lamina of the blebs appears as a complete circle, distinct from the nucleus. There are strong examples of LB1+ blebs from fixed cells in Fig. 2A (WT MEF, left) and Fig. 4A (DU145), demonstrating that blebs can be distinguished from micronuclei with their imaging parameters.

Authors' response: In summary, this reviewer believes:
1) That nuclear blebs cannot have a fully encased or a complete circle of nuclear lamina.
2) The blebs containing high levels lamin B1 are not blebs but micronuclei.
3) That lamin B1 loss is uniform in live cells and non-uniform in fixed cells.
4) The reviewer states: "**The finding that DNA density in blebs is consistently reduced is strong enough to merit publication of this paper…**"

We directly address these statements:
1) **New data in revised Figure 6C** clearly shows that time-lapse verified nuclear blebs can have lamin B1 encased nuclear blebs, which is also seen in a different cell type in **Figure 3**. Thus, nuclear blebs can have a nuclear bleb fully incases in lamin B1.
2) **New data in Supplemental Figure 1A** show that live cell imaging of prostate cancer cell

© 2024. Published by The Company of Biologists under the terms of the Creative Commons Attribution License (https://creativecommons.org/licenses/by/4.0/).

line LNCaP have very rare micronuclei < 0.6% (2/326 cells). Thus, LNCaP nuclear blebs with similar/high lamin B1 bleb to body ratio (Figure 4), it is impossible to explain this phenomenon by confusion of a bleb for a micronucleus because there are 4-5 fold less micronuclei than blebs.

3) **Original data in the manuscript Figure 3** shows that live cell and fixed cell imaging are similar, refuting the reviewer's statement.
4) **We agree that the title and main finding of the paper is the DNA density is a better marker of nuclear blebs.** We have provided new data in revision #1 and now revision #2 to address changes in lamin B1 levels in nuclear blebs, even though this is not the focus of the paper – echoed by this reviewer.

1) This assertion that a nuclear bleb cannot be encased in lamins is based on no data and likely stems from older qualitative data that suggested lamin B1 is absent in a nuclear bleb. Original manuscript data via live cell imaging of HCT116 shows that newly formed nuclear blebs can be fully encased in lamin B1 (**Figure 3**) <u>and now we provide new data</u> showing the same for MEF live cell into immunofluorescence (**Figure 6C**).

In the first revision of the manuscript, we used live cell imaging to determine how often a micronucleus touches the nuclear body:

- 9% chance - measured by time-lapse, the chance a micronucleus touches the nucleus
- 4.5% - half of the micronuclei touching the nucleus can be ruled out by a Hoechst intensity greater than 0.8 of the nuclear body (**Figure 1E**).
- < 4.5% - not all micronuclei have lamin B1 staining
- < 4.5% micronuclei vs. measured 29% of nuclear blebs with lamin B1 via immunofluorescence AND 22% of nuclear blebs with lamin B1 via live cell imaging into immunofluorescence **(Figure 1** vs **Figure 2 and 6**). <u>To be clear, live cell imaging provides a means to distinguish with the highest certainty a nuclear bleb from a micronucleus</u> (**Figure 1 and 6**) and thus time lapse imaging into fixed cell immunofluorescence would reveal the true behavior of lamin B1in a nuclear bleb, which we show can encase a bleb (**Figure 6C**).

Thus, in fixed cell immunofluorescence the chance that we are confusing a bleb for a micronucleus is rare <4.5%. Furthermore, this low rate would have to account for the around 25% of lamin B1 high nuclear blebs. Which the data shows are not logically possible when time-lapse verification into immunofluorescence provides a similar number of blebs containing lamin B1 at 22%.

Specfically, we believe the reviewer expects nuclear blebs to have a clear "neck" connecting the nucleus with the bleb where no lamina exists. This idea of "neck" comes from nuclear blebs that loose the lamina during nuclear rupture. The reviewer has requested z stack imaging to see this "neck". We have added new data to Figure 6 as panel C showing that a time-lapse verified nuclear bleb can be encased in lamin B1. This new data direct addresses the reviewers point that : "[fixed cell nuclear blebs encased in lamin B1 are] **consistent with micronuclei as the nuclear lamina of the blebs appears as a complete circle, distinct from the nucleus."** In fact, nuclear blebs can have this behavior as our data shows, refuting the reviewers point. Specifically, The z stack shows that the majority ¾ of in focus images show a bleb encased in lamin B1. However, 1 image of the 4 does show a "neck" connecting the bleb to the nuclear body void of lamin B1, though the DNA signal is out of focus. This data supports two findings: 1) this lamin B1 containing bleb does show connected to the body and 2) that most images show a fully encased lamin B1 bleb, which is what other researchers will mostly see when they do the same analysis.

Why does the nuclear bleb appear encased or encircled in lamin B1 if it is connected to the nucleus? Unpublished data we have for a different manuscript shows the connection between the nuclear body and bleb, we call the neck, is narrow in that it has low height. This lower height and thin x,y means that the lamin is visualized as encircling the bleb in most focal planes.

NOTE: We have removed unpublished data that had been provided for the referees in confidence.

Unpublished Figure from a different manuscript. Height measurements confirm constriction between the nuclear body and bleb. Example confocal images of a Hoechst- stained blebbed nucleus (A) viewed top down (x,y) at 1 um focal plan increments, (B) side view slice denoting body (gray), neck (magenta), and bleb (yellow), and (C) 3D view. (D) Graph height of body (gray), neck (magenta), and bleb (yellow) measured by full-width half-maximum of fluorescence

© 2024. Published by The Company of Biologists under the terms of the Creative Commons Attribution License (https://creativecommons.org/licenses/by/4.0/).

signal from side view. (E) Graph of each single nucleus body, neck, and bleb. Confocal images were taken at 200 nm z-steps. Statistical significance noted by asterisks ** $p < 0.01$ and ** $p < 0.001$. Scale bar = 10 μm.

Reviewer's comments continued:
The current data strongly suggest that decreased DNA concentration is a hallmark of nuclear blebs, but the conclusions about LB1 variability on blebs between conditions and disease models are still not adequately supported. The alternative hypothesis, that these conditions alter micronucleation frequency and/or appearance and that these are the major source of high LB1 levels in "blebs", is equally likely based on the representative images.

Authors' response:
2) The reviewer suggests that lamin B1 variability could be due to increased micronucleation frequency and confusion of those micronuclei for blebs. <u>Our newly added data analysis refutes this concern</u>. hTERT WT and Progeria present low lamin B1 levels in nuclear blebs while prostate cancer cells show high levels (**Figure 4**). In the revised manuscript we measured the frequency of micronucleation in LNCaP cells via time-lapse imaging because these cells showed majority of nuclear blebs have high lamin B1 levels, as well as all prostate cancer cell lines. We report 1 micronucleus per biological replicate imaging 187 and 170 cells, representing an average micronucleation frequency of 0.6% compared to a blebbing percentage of 2.7%. Thus, nuclear blebs occur 4.5-fold more than micronuclei. This rate is similar for fixed cells as we found 10 micronuclei for 40 nuclear blebs, a 4-fold difference. Similarly, fixed cells with another prostate cancer cell line PC3 resulted in only 4 micronuclei present while we measured 63 nuclear blebs, of which 34 (53%) showed no change in lamin B1 levels in the bleb classified as 0.9 or greater relative to the body (**Figure 4**). <u>PC3 and LNCaP high lamin B1 nuclear blebs could not possibly be due to confusion of micronuclei for nuclear blebs simply because there are not enough micronuclei to confuse for nuclear blebs</u>.
Alternatively, the data supports our hypothesis that loss or maintenance of lamin B1 levels in the nuclear bleb are due to rupture or not, respectively. Nuclear blebs verified by timelapse, can form with or without normal levels of lamin B1 (**Figure 3**) and can have lamin B1 normal or decreased levels dependent on nuclear rupture (**Figure 6**). Thus, lamin B1 variability is not due to confusion of micronuclei as a bleb but instead is a clear property of nuclear blebs that appear to stem from nuclear rupture or lack thereof.

Reviewer's comments continued:
The most compelling evidence that decreased DNA density is a consistent maker of blebs is the HCT116 GFP-B1 live cell imaging analysis where multiple criteria are used to identify blebs. However, in these data LB1 is uniformly depleted in blebs (Fig. 3B), unlike in the analysis of fixed cells (Fig. 3A), suggesting that the fixed cell criteria are including non-blebs in the analysis.
Authors' response:
3) The reviewer thinks that LB1 is uniform in Fig 3B (live cell) and not in Fig 3A (fixed cell). This statement lacks data to back it as LB1 levels are a spectrum between 0.4 and 1.2 in 3A and 0.3 and 1.1 in 3B. It is possible the reviewer is commenting on how in Figure 3A the lamin B2 (LB2, purple) is less uniform than LB1? Though this is just an example image – so it is very unclear what the reviewer is asking.
The point of Figure 3 is to show that lamin B1 levels can be high (similar to body) or significantly decreased. The most powerful piece of data is that a nuclear bleb can be seen forming with normal/high levels of lamin B1 or loss of lamin B1 (**Figure 3C**). <u>This newly formed bleb is also encased in lamin B1.</u> Taken together live cell imaging to verify a nuclear bleb in both MEF and HCT116 reveal that nuclear blebs can have variable levels of lamin B1. In the past revision we provided data that showed these lamin B1 normal/high containing blebs were due to lack of rupture, while loss of lamin B1 occurred in nuclei that rupture within 1 hour of immunofluorescence (**Figure 6**).
We would like to highlight that the reviewer strongly agrees with the title of the paper – that DNA is a better marker of nuclear blebs. They clearly state here:
"The most compelling evidence that decreased DNA density is a consistent maker of blebs is the HCT116 GFP-B1 live cell imaging analysis where multiple criteria are used to identify blebs."

© 2024. Published by The Company of Biologists under the terms of the Creative Commons Attribution License (https://creativecommons.org/licenses/by/4.0/).

Reviewer's comments continued: The finding that DNA density in blebs is consistently reduced is strong enough to merit publication of this paper and I am concerned this will get lost in disagreements about what is being analyzed.
Authors' response: The reviewer acknowledges that the core data are strong enough for publication. We have done everything we can scientifically to address their concerns about lamin B1 variability.
Reviewer's comments continued: Therefore, I strongly recommend the authors reanalyze their fixed images using the more conservative criterium of NL connectedness to distinguish blebs from micronuclei and report the results.
Authors' response: It is completely unreasonable to suggest that we reanalyze with "more conservative criterium". We have provided clear criterium for determining a nuclear bleb and shown that it is aligned with live cell studies (**Figure 1, 3, 6**) and different approaches (**Figure 5**). We have addressed the reviewer's alternative hypothesis of micronuclei confusion, increased micronucleation, and claim that lamin B1 "cannot" encase a nuclear bleb. We have shown all of these to be refuted by the data. Alternatively, we have provided many new pieces of data since the original submission to support our conclusions.
Reviewer's Minor comments:

1. **Decreasing the point opacity on the dot plots would improve readability.**
   The dot plot is to provide overall spread while the raw data is available in the supplemental material.
2. **Fig. 2B - add stats to graph.**
   There is no statistical significance change ($p > 0.05$) and thus no asterisks which is noted in the figure legend.
3. **Fig. S1A - these data are hard to interpret without the nuclear bleb intensity values on the same graph, especially for the prostate cancer cell lines.**
   Data cannot be posted in two places in the manuscript via JCS guidelines.
4. **The description of the causal link between LB1 in the bleb and bleb rupture is inconsistent between the results and the discussion. The results section implies that rupture causes a loss of LB1 whereas the discussion implies that LB1- blebs have an increased likelihood of rupture. The second statement is strongly supported by the literature, whereas the first one requires additional experiments to validate. The results section should therefore be modified to match the discussion section.**

We thank the reviewer for bringing our attention to this writing error and have revised the text. "we would hypothesize the lack of lamin B1 loss in the bleb relative to the body ~~should result in~~ is due to lack of bleb rupture. Similarly, we would hypothesize both hTRET and NGPS progeria nuclear blebs rupture frequently ~~because they~~ causing them to present low lamin B1 levels in the nuclear bleb. Oppositely, we would hypothesize that prostate cancer cells lines' nuclear blebs rupture infrequently ~~due to~~ which results in their high levels of lamin B1 in the bleb (Fig. 4)."

*****

Reviewer 2 Advance Summary and Potential Significance to Field:
This is a second round of review of an interesting work addressing the question of the composition of the nuclear blebs, an important point in cell biology.

Reviewer 2 Comments for the Author:
I would like to thank the authors for considering my concerns and providing a revised version of their manuscript according to the Reviewers' comments.
The authors satisfactorily replied to most of my comments and I think that the manuscript has much improved, however I still have some concerns indicated below.

Major comments:

1) **About the point of "How reliable are DNA fluorescence markers DAPI, Hoechst, SiR-DNA" the authors added some experiments quantifying DAPI fluorescence relative to cell cycle phases markers (geminin/cdt1) and replied that "We and many others have previously published that increased DNA content changes during the cell cycle by these DNA markers, as the total fluorescence intensity doubles from G1 to G2. We have included new data in the revised manuscript showing that these DNA dyes provide quantitative data on DNA amount. HT1080 FUCCI cells that provide fluorescence reporters for G1 cells which have diploid genome vs late S/G2 cells that have tetraploid**

© 2024. Published by The Company of Biologists under the terms of the Creative Commons Attribution License (https://creativecommons.org/licenses/by/4.0/).

genome. In short, Hoechst measured DNA content nearly doubles from the G1 to G2, as expected (Supplemental Figure 1 B-E).”

It is true that if we consider G1 vs lateS-G2 DNA nuclear content can be estimated by DAPI signal. This is straightforward since the content is doubled in these two different moments of the cell cycle. However, the authors here are not quantifying DNA of the cells that are going through cell division. And even for cell cycle analysis by flow cytometry DAPI is not frequently used because it preferentially stains AT-rich DNA thus underestimating DNA content. The authors, here, would like to use DAPI (or siRDNA or HOECHST) fluorescence in IF to measure the different DNA content between nuclear body and bleb within the same nucleus in whatsoever cell cycle phase. DAPI staining, as it is clearly noticed also in the manuscript figures, generally does not stain the nucleus in a homogeneous way, it depends on the level of DNA condensation. This is not taken into account in the manuscript nor in the authors’ reply and I am afraid that this could be an essential issue in the context. Considering the delicacy of the matter and the importance of this data, I would rather use the term DNA density or better DAPI signal instead of DNA amount/concentration/quantity/content.

Accordingly, the authors should rephrase this part of the manuscript and the following claims based on this type of quantification and in the abstract.

2) Related to 1) the authors did not provide a comparison with another more proper DNA staining such as Propidium Iodide as suggested in the first review. The authors replied that DAPI staining is as quantitative as PI, however they show published paper about flow cytometry analysis which is way different from microscope images analysis. Comparing with staining with FUCCI system only tells the phase of the cell cycle and not the exact DNA content in the analysed conditions.

1) We appreciate the feedback and have made the suggested revision throughout the manuscript to use the term DNA density.
2) In the revised manuscript we provide new data showing that propidium iodide (PI) staining is similar to DAPI/Hoechst. We report that PI relative intensity compared to the nuclear body decreased in the nuclear bleb while micronuclei show increased relative intensity (**Supplemental Figure 1G**). Given all of the supporting data, including PWS data, we have provided multiple methods to measure decreased DNA density in the nuclear bleb. Furthermore, we would like to highlight reviewer #1’s statement, “The finding that DNA density in blebs is consistently reduced is strong enough to merit publication of this paper..”.

Minor Comment:

-Authors’ reply:” C) The statement that “Quantification heterochromatin markers would be more informative…” does not fall in the scope of the paper. We are investigating DNA concentration, not chromatin histone modification state.

Furthermore, heterochromatin is composed of many specific histone modifications (H3K9me3, H3Kme2, H3K27me3 to name a few) and thus would require an entire other manuscript to address this suggested approach making it clearly beyond the scope of the current manuscript.”

I understand that checking the epigenetic markers could have been dispersive and partially repetitive due to already published papers on the matter, however, the authors only provide DAPI staining to quantify DNA concentration and, again, DAPI staining quantification by IF is not a fine methodology to quantify DNA.

As stated in the above response, we now included new data showing that PI staining is also decreased in the nuclear bleb relative to the nuclear body (**Supplemental Figure 1G**).

*****

Reviewer 3 Advance Summary and Potential Significance to Field:

The submitted manuscript presents data indicating that a reduction in chromatin staining intensity is a more consistent indicator of nuclear blebs than a reduction in lamin b1 intensity. This will be a useful criterion in the identification of blebs and can also provide mechanistic insight into their organization and function.

We thank the reviewer for their acknowledgement of the significance of our work.

Reviewer 3 Comments for the Author:

The authors have addressed all my concerns and have now carried out all the

© 2024. Published by The Company of Biologists under the terms of the Creative Commons Attribution License (https://creativecommons.org/licenses/by/4.0/).

appropriate statistical and editorial modification that I suggested.

Third decision letter

MS ID#: JOCES/2024/262082

MS TITLE: Decreased DNA density is a better indicator of a nuclear bleb than lamin B loss

AUTHORS: Samantha Bunner, Kelsey Prince, Emily M Pujadas Liwag, Nebiyat Eskndir, Karan Srikrishna, Pedro Pellegrino, Julianna Aiello, Antonia Amonu McCarthy, Colman Lawlor, Shrushti Jagtap, Anna Kuklinski, Emma Eastin, Griffin Yas, Nathan LaPointe, Olivia Jackson, Imuetiyan Eweka, Isabelle Schramm von Blucher, Jillian Hardy, Jason Chen, Schuyler Figueroa, Vadim Backman, Anne Janssen, Mary Packard, Katherine Dorfman, Luay Almassalha, Michael Seifu Bahiru, and Andrew D Stephens

I am happy to tell you that your manuscript has been accepted for publication in Journal of Cell Science, pending standard publication integrity checks.

© 2024. Published by The Company of Biologists under the terms of the Creative Commons Attribution License (https://creativecommons.org/licenses/by/4.0/).
